# CD81 Receptor Regions outside the Large Extracellular Loop Determine Hepatitis C Virus Entry into Hepatoma Cells

**DOI:** 10.3390/v10040207

**Published:** 2018-04-20

**Authors:** Pia Banse, Rebecca Moeller, Janina Bruening, Lisa Lasswitz, Sina Kahl, Abdul G. Khan, Joseph Marcotrigiano, Thomas Pietschmann, Gisa Gerold

**Affiliations:** 1 Institute for Experimental Virology, TWINCORE, Centre for Experimental and Clinical Infection Research, 30625 Hannover, Germany; pia.banse@googlemail.com (P.B.); rebecca.moeller@twincore.de (R.M.); janina.bruening@googlemail.com (J.B.); lisa.lasswitz@twincore.de (L.L.); sina.kahl@icloud.com (S.K.); thomas.pietschmann@twincore.de (T.P.); 2 Center for Advanced Biotechnology and Medicine, Department of Chemistry and Chemical Biology, Rutgers University, Piscataway, NJ 08854, USA; akhan@tritdi.org (A.G.K.); joseph.marcotrigiano@nih.gov (J.M.)

**Keywords:** hepatitis C virus, HCV, tetraspanin, CD81, receptor, chimeras, susceptibility-determining domains, transmembrane domain four, cholesterol-binding residue

## Abstract

Hepatitis C virus (HCV) enters human hepatocytes using four essential entry factors, one of which is human CD81 (hCD81). The tetraspanin hCD81 contains a large extracellular loop (LEL), which interacts with the E2 glycoprotein of HCV. The role of the non-LEL regions of hCD81 (intracellular tails, four transmembrane domains, small extracellular loop and intracellular loop) is poorly understood. Here, we studied the contribution of these domains to HCV susceptibility of hepatoma cells by generating chimeras of related tetraspanins with the hCD81 LEL. Our results show that non-LEL regions in addition to the LEL determine susceptibility of cells to HCV. While closely related tetraspanins (*X. tropicalis* CD81 and *D. rerio* CD81) functionally complement hCD81 non-LEL regions, distantly related tetraspanins (*C. elegans* TSP9 amd *D. melanogaster* TSP96F) do not and tetraspanins with intermediate homology (hCD9) show an intermediate phenotype. Tetraspanin homology and susceptibility to HCV correlate positively. For some chimeras, infectivity correlates with surface expression. In contrast, the hCD9 chimera is fully surface expressed, binds HCV E2 glycoprotein but is impaired in HCV receptor function. We demonstrate that a cholesterol-coordinating glutamate residue in CD81, which hCD9 lacks, promotes HCV infection. This work highlights the hCD81 non-LEL regions as additional HCV susceptibility-determining factors.

## 1. Introduction

Virus infection of cells is initiated by interaction with cell surface receptors. Receptors bind virus particles and trigger an uptake program, which can include receptor clustering, lateral membrane movement, signaling, internalization, trafficking, membrane penetration and uncoating [1]. Mechanistically, protein–protein interactions (PPI) and protein–lipid interactions guide these steps [2,3,4,5]. While the receptors for some viruses are known, we typically lack knowledge on the post-binding function of virus receptors, i.e., how they mechanistically induce virus uptake.

Hepatitis C virus (HCV) is a member of the *Flaviviridae* family and as such a small enveloped positive strand RNA virus. It is the causative agent of chronic hepatitis C, which affects approximately 71 million individuals worldwide [6,7]. HCV uses four essential entry factors: scavenger receptor type B class I (SR-BI), human CD81 (hCD81), claudin-1 (CLDN1) and occludin (OCLN) [8,9,10,11]. A second non-canonical uptake pathway using very low density lipoprotein receptor (VLDLR) and LDLR instead of SR-BI exists and presumably relies on the HCV particle associated serum lipoproteins [12,13]. In contrast, SR-BI and hCD81 bind HCV particles directly through the viral E2 glycoprotein [8,11]. In addition, hCD81 mediates post-binding steps in HCV entry. It translocates with the virus along the plasma membrane [14], it co-endocytoses with the virus [15] and it triggers conformational changes in the viral E1/E2 glycoproteins rendering them competent for fusion with the endosomal membrane [16]. Which domains of hCD81 are required for these post-binding functions remains largely elusive.

CD81 belongs to the tetraspanin protein family. It is surface expressed and consists of four transmembrane domains, two short cytoplasmic termini, one short cytoplasmic loop and two extracellular domains, termed the small extracellular loop (SEL) and the large extracellular loop (LEL). Tetraspanins lack signaling domains, but coordinate PPIs and lipid interactions in tetraspanin webs [17,18,19,20]. Human CD81 associates with the HCV entry factors SR-BI and CLDN1 [21,22] and co-factors, such as the GTPase HRas and serum response factor binding protein 1 (SRFBP1) [22,23]. Thus, hCD81 has at least two functions during HCV entry. First, it concentrates HCV at the cell surface. Second, it engages in interactions with host proteins and lipids needed for post-binding entry steps. For the former, the structure–function relationship of hCD81 has been extensively analyzed [8,24,25]. Specifically, the LEL region of hCD81 directly interacts with HCV E2 glycoprotein. In contrast, the structural requirements of hCD81 to engage in protein and lipid interactions needed for HCV entry are poorly defined. To function as a HCV receptor, CD81 needs to dynamically move in the plasma membrane, possibly to engage in different molecular interactions during virus penetration [26]. Some interactions of hCD81 with host proteins such as immunoglobulin superfamily member 8 (IGSF8) map to regions outside the LEL, here referred to as backbone [27]. In addition, transmembrane domains one and four form hydrogen bonds with cholesterol [19]. Thus, the hCD81 backbone (intracellular tails, four transmembrane domains, small extracellular loop and intracellular loop) can coordinate interactions with lipids and proteins, some of which may be required for HCV cell entry.

Importantly, hCD81 is one of the species tropism determining factors for HCV [24]. The virus naturally infects humans and can experimentally infect chimpanzees and tupaias [28,29,30]. A combination of host factor incompatibility and innate immune restriction leads to this narrow host tropism [31,32,33]. Murine and rat CD81 inefficiently function as HCV receptors and expression of hCD81 together with human OCLN renders mice susceptible to HCV [32,34]. In vitro studies demonstrate that the murine CD81 LEL cannot bind HCV E2 efficiently [35]. Mouse CD81 backbone domains, however, when fused to the hCD81 LEL function as HCV receptor [24]. Therefore, CD81 backbone functions are conserved between human and rodent orthologs. Whether any tetraspanin backbone from more distantly related species than rodents could substitute the hCD81 backbone function in HCV entry remains largely undefined.

Here, we set out to investigate whether regions of hCD81 outside the LEL are critical determinants of HCV entry. Therefore, we constructed and characterized chimeric tetraspanins composed of the hCD81 LEL and backbone domains (intracellular tails, transmembrane domains, small extracellular loop and intracellular loop) of related tetraspanin molecules (orthologs and paralogs). Three out of seven tested chimeras can bind HCV E2 glycoprotein, but fail to support HCV cell entry. In particular, backbones from distantly related tetraspanins such as the *Drosophila melanogaster* (dm) ortholog and hCD9 fail to function as efficient HCV receptors. While for some chimeras reduced surface expression correlates with reduced receptor function, the dm and hCD9 chimeras are surface expressed, bind HCV E2 glycoprotein, but fail to support HCV infection. Specifically, we identified the E219 residue in transmembrane domain four of hCD81, which coordinates cholesterol and is lacking in hCD9, to promote HCV infection. Together, these data highlight the critical role of hCD81 backbone domains in post-binding HCV entry events.

## 2. Materials and Methods

### 2.1. Tetraspanin Chimera Construct Generation

Chimeric tetraspanins were designed by replacing the LEL of human CD9 (hCD9), human CD82 (hCD82), human TSN32 (hTSN32), *Danio rerio* CD81 (drCD81), *Xenopus tropicalis* CD81 (xtCD81), *Caenorhabditis elegans* TSP9 (ceTSP9) and *Drosophila melanogaster* TSP96F (dmTSP96F) with the hCD81 LEL (aa 113–201). The Uniprot annotation [36] of transmembrane domains 3 and 4 was confirmed by transmembrane domain prediction (DAS-TM filter [37]) and used to determine the LEL insertion site. All constructs contained a tandem hemagglutinin (HA) tag fused to the C-terminus by a Gly_4_SerGly linker. Chimeric tetraspanin cDNAs were synthesized flanked by BamHI and SpeI or SbfI and SpeI restriction sites and amplified by PCR using the primers: dr/xtCD81-f (5′-AAA AAA GGA TCC GCC ACC ATG GG-3′), ceTSP9-f (5′-AAA AAA GGA TCC GCC ACC ATG GTG-3′), dmTSP96F-f (5′-AAA AAA CCT GCA GGG CCA CCA TGG GTC TCA ACG-3′), hCD9-f (5′-AAA AAA CCT GCA GGG CCA CCA TGG GTC TCA ACG-3′), hCD82-f (5′-AAA AAA CCT GCA GGG CCA CCA TGG GCT CAG C-3′), hTSN32-f (5′-AAA AAA CCT GCA GGG CCA CCA TGG GGC CTT G-3′) and HA-r (5′-AAA AAA ACT AGT CTA GGC GTA GTC GG-3′). PCR products were cloned into the pWPI_BLR lentiviral vector using the BamHI or SbfI and SpeI restriction sites and inserts confirmed by sequencing. E219 mutants were cloned by fusion PCR using primers carrying the respective mutated codon: E219A-f (5′-TCG CGA TGA TCC TGA GCA TGG TGC TG-3′), E219A-r (5′-CAT GCT CAG GAT CAT CGC GAA GAT CAT GAT CAC AGC GAC C-3′), E219Q-f (5′-TCC AGA TGA TCC TGA GCA TGG TGC TG-3′), E219Q-r (5′-CAT GCT CAG GAT CAT CTG GAA GAT CAT GAT CAC AGC GAC C-3′), E219S-f (5′-TCT CGA TGA TCC TGA GCA TGG TGC TG-3′), E219S-r (5′-CAT GCT CAG GAT CAT CGA GAA GAT CAT GAT CAC AGC GAC C-3′) and the flanking primers HAHA-Gibson-r (5′-TCC TGC AGC CCG TAG TTT AC-3′) and hCD81-Gibson-f (5′-TTA AAC CTG CAG GCG CGC CG-3′). PCR products were cloned into the pWPI_BLR vector by Gibson assembly according to the manufacturer’s instructions (New England Biolabs, Ipswich, MA, USA).

### 2.2. Cell Culture, Ectopic Expression of Tetraspanin Variants in Lunet N#3 Cells and CHO745 Cells, HCV Replicons, HCV Pseudoparticles, and Cell Culture-Derived HCV (HCVcc)

The human hepatoma cell lines Huh-7.5 [38], which expresses hCD81, and Lunet N#3, which lacks detectable levels of endogenous hCD81 [39], were cultured in Dulbecco’s Modified Eagle’s Medium (DMEM) supplemented with 10% fetal bovine serum, 2 mM glutamine and non-essential amino acids at 37 °C. CHO745 cells [40], which lack glycosaminoglycans and hCD81, were cultured in RPMI 1640 supplemented with 10% fetal bovine serum, 2 mM glutamine and 10 mM Hepes at 37 °C. Vesicular stomatitis virus (VSV) pseudotyped lentiviral particles with tetraspanin chimera and point mutant encoding proviral pWPI plasmids with blasticidin resistance were generated by triple transfection of HEK 293T cells. Lunet N#3 and CHO745 were subsequently transduced with lentiviral particles for 6 h at 37 °C [39]. Lunet N#3 and CHO745 cells expressing tetraspanin chimeras and point mutants were stably selected using blasticidin. Luciferase- and GFP-based HCV pseudoparticles were generated as described [41,42]. Jc1, Jc-R2A and intergenotypic chimera HCVcc were produced in Huh-7.5 cells and titrated as previously reported [23].

### 2.3. Immunoblotting

Cell lysates were prepared from scraped cells after extensive PBS washes in Hepes buffer (50 mM Hepes, pH 7.4, 150 mM NaCl, 10% glycerol, 1% NP-40, 1 mM CaCl_2_) containing protease inhibitors at a dose recommended by the manufacturer (Sigma, St. Louis, MO, USA, P8340). Total protein concentration was measured by Bradford assay, and SDS-PAGE and immunoblotting of 50 μg cell lysates were performed as described in [43].

### 2.4. Immunofluorescence Analysis and Confocal Microscopy

Cells were grown on poly-(l)-Lysine coated cover slips at a density of 4.5 × 10^4^ cells per 24-well for 24 h, fixed with 4% paraformaldehyde, permeabilized for 4 min with 0.1% Triton X-100 in PBS, blocked for 10 min with 0.5% BSA in PBS and stained with mouse anti-hCD81 (JS-81, BD Biosciences, San Jose, CA, USA, 0.2 μg/mL) or rabbit anti-HA (H6908, Sigma Aldrich, St. Louis, MO, USA, 0.5 μg/mL) and mouse anti-p230 (clone 15, BD Biosciences, 1.25 μg/mL) over night at 4 °C in PBS with 0.5% BSA. Staining with secondary antibodies (goat anti-mouse-IgG-Alexa488 and goat anti-rabbit-IgG-Alexa647, 2 μg/mL, Life Technologies, Carlsbad, CA, USA) was performed for 1 h at room temperature. Nuclei were counterstained with DAPI (300 nM, Life Technologies) and coverslips mounted in Prolong Gold (Life Technologies) followed by inverse confocal laser-scanning microscopy (Olympus Fluoview 1000), using ×60 and ×100 magnification lenses. Channels were read in sequential acquisition mode with a Kalman filter (*n* = 3) and single planes of at least five frames per biological replicate were analyzed.

### 2.5. CD81 Surface Staining and Flow Cytometry

Cells were surface stained in PBS supplemented with 1% FCS using anti-hCD81-FITC (BD, JS-81, 20 μL per test), anti-hCD81-APC (BD, JS-81, 20 μL per 1 × 10^6^ cells in 200 μL final volume), anti-hCD81 (Santa Cruz lnc., Santa Cruz, CA, USA, 5A6, 1 μg/mL), or isotype controls (BD, 20 μL per test for fluorophore conjugates or 1 μg/mL for unconjugated antibodies) for 20 min on ice. For unconjugated primary antibodies, anti-mIgG-PE (eBioscience, Waltham, MA, USA) was used as secondary antibody at 1 μg per 1 × 10^6^ cells. After staining, cells were washed three times with PBS supplemented with 1% FCS and analyzed by flow cytometry (BD Accuri).

### 2.6. Soluble E2 Binding Assay

E2 ectodomains (eE2) were expressed and purified as described previously [44]. E2 was labeled with Alexa-488 or Alexa-647 according to the manufacturer’s instructions using the Alexa Fluor 488 Antibody Labeling Kit or the Alexa Fluor 647 Antibody Labeling Kit (Molecular Probes, Waltham, MA, USA). Then, 5 × 10^6^ CHO 745 cells were incubated with 10 μg of Alexa fluorophore conjugated eE2 in 600 μL PBS at room temperature for 2 h. Cells were washed three times with PBS and analyzed by flow cytometry (BD Accuri).

### 2.7. Pseudoparticle Infection

Cells were transduced with lentiviral pseudoparticles as previously described [23]. For flow cytometric infectivity readout, the GFP encoding lentiviral proplasmid pWPI.Venus.GFP was used and cells were counterstained with anti-hCD81-APC at 72 h post transduction (hpt) before flow cytometric analysis.

### 2.8. HCVcc, VSV and Coronavirus Infection

Cell culture infectious HCV (Jc1, JcR2A and intergenotypic chimeras) infection and JFH-1 subgenome replication assays were performed as described [23]. Briefly, in vitro transcribed HCV genomes were electroporated into Huh-7.5 cells. Cell culture HCV was harvested 48 h, 72 h and 96 h post electroporation and used to infect hepatoma cells. Infectivity was determined by luciferase activity measurement in cell lysates at 72 h post infection (hpi). Subgenomic replicon transcripts were electroporated into hepatoma cells and luciferase activity was measured at indicated timepoints. VSV*M_Q_ and HCoV229-E-luc were kindly provided by Gert Zimmer [45] and Volker Thiel [46], respectively, and cells infected as reported in [23]. Briefly, hepatoma cells were seeded at a density of 8 × 10^3^ in 96-wells and infected with an MOI of 0.1 of VSV*M_Q_ and HCoV229-E-luc 24 h later. VSV*M_Q_ infectivity was assessed 16 hpi by flow cytometric quantification of GFP positive cells. HCoV229-E-luc infectivity was determined by luciferase activity measurement in cell lysates at 24 hpi as described in [43].

### 2.9. HCV RNA Quantification

HCV JFH-1 subgenomic RNA was quantified in cellular extracts 4 h and 48 h post electroporation using established protocols and Taqman probes (Applied Biosystems, Foster City, CA, USA) with GAPDH as internal reference [23].

### 2.10. HCV Plasma Membrane Fusion Assay

To assay for endosomal acidification independent HCV uptake, we treated cells with 5 nM concanamycin A to prevent acidification of endosomal compartments and bound JcR2A to the cells for 2 h at 4 °C. Membrane fusion was induced by a 5 min wash with pH 5 citric acid buffer. Background infection was assessed by a 5 min wash with pH 7 citric acid buffer. Infectivity was measured as luciferase activity at 48 hpi. Please refer to [23] for a detailed description.

### 2.11. Alignments, Computational and Statistical Analyses

Amino acid alignments and percentage identity calculations were performed using the EMBL-EBI Clustal Omega algorithm [47]. Pairwise distances were calculated in SSE (version 1.2, Oxford, OX1 3SY, UK) with a numeric precision of 4, a fragment length of 24 and an increment between fragments of 9 [48]. Hydrophobicity analysis according to Kyte–Doolittle was performed using CLC Genomics Workbench (Qiagen, Hilden, Germany). The hCD81 structure (PDB:5TCX) was imaged with 3-D Molecule Viewer (Invitrogen, Carlsbad, CA, USA, Vector NTI Advance 11.0). Exponential growth regression analysis to correlate sequence identity and susceptibility as well as linear regression analysis of tetraspanin surface expression and HCV susceptibility were performed in GraphPad Prism 5 (GraphPad Software, Inc., San Diego, CA, USA).

Experiments were performed at least in three biological replicates with three technical replicates per experiment unless otherwise stated. Results are presented as mean plus the standard deviation (SD) of three biological replicates unless otherwise indicated. The 50% tissue culture infectious dose (TCID50) was calculated based on Kaerber and Spearman [49,50]. Statistical significance was determined by one-way Analysis of Variance (ANOVA) followed by Dunnett’s multiple comparison test in GraphPad Prism 5 (GraphPad Software, Inc., San Diego, CA, USA). A *p* value of less than 0.05 was considered statistically significant.

## 3. Results

### 3.1. CD81 Backbone Chimeras Bind to HCV E2 Glycoprotein

**Figure 1:**
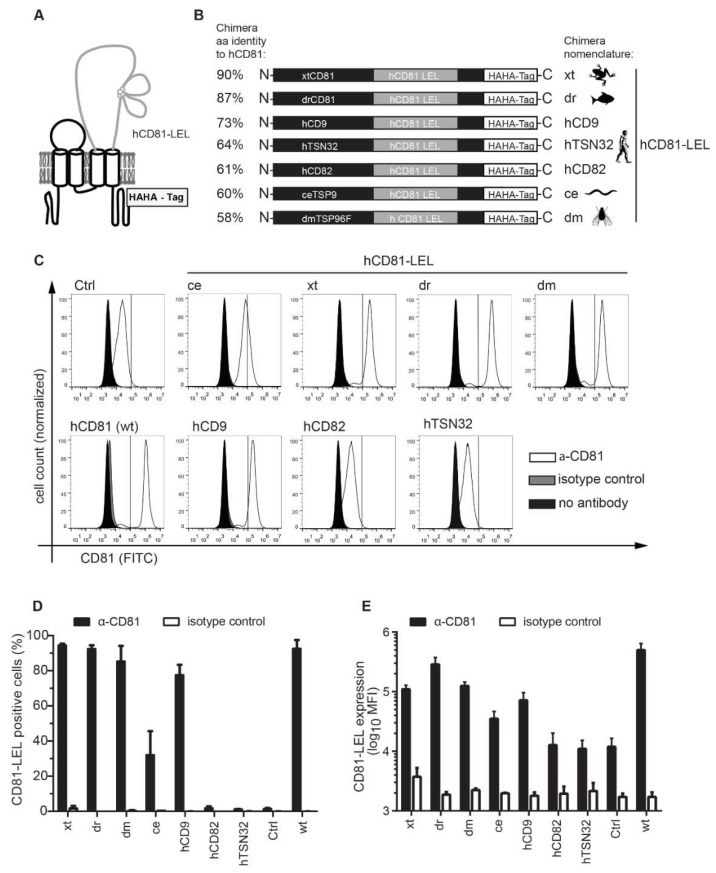
Expression of hCD81 chimeras on CHO745 cells. (**A**) Schematic representation of the hCD81 chimeras used in this study; (**B**) Amino acid sequence identities calculated by Clustal W and domain structure of the chimeras; (**C**) Flow cytometric analysis of surface expression on CHO745 cells. Cells were transduced with the indicated hCD81 chimeras, full-length hCD81 (wt) or empty vector (Ctrl) and selected with blasticidin. CD81 surface expression was determined by staining with an antibody directed against the hCD81 LEL (clone JS-81). The histograms show cells stained with anti-hCD81, an isotype control antibody or cells left unstained. Vertical lines indicate gating for CD81 positive cells; (**D**) Quantitation of hCD81 surface staining depicted as percentage of positive cells or (**E**) as mean fluorescence intensity (MFI). Mean values + SD of three independent biological replicates are shown in (**D**,**E**). The histograms show one out of three representative experiments with 30,000 cells per measurement.

To determine the contribution of hCD81 backbone domains to HCV entry, we generated chimeras of hCD81 LEL with backbones from the human tetraspanins hCD9 (45% amino acid sequence identity with hCD81), hCD82 (26%) and hTSN32 (15%) as well as from the hCD81 orthologues in *Xenopus tropicalis* (xt, 72%), *Danio rerio* (dr, 64%), *Drosophila melanogaster* (dm, 27%) or *Caenorhabditis elegans* (ce, 25%) (Figure 1A and Table 1). The tetraspanins’ Uniprot annotation as well as Kyte–Doolittle hydrophobicity analysis served to determine the transmembrane domains 3 and 4, between which we inserted the hCD81 LEL (aa 113 to 201 of hCD81) (Figure 1B). The resulting hLEL tetraspanin chimeras were named according to the origin of the respective tetraspanin backbone domain (e.g., xt for the hCD81 LEL–xtCD81 backbone chimera) and displayed amino acid sequence percent identities to parental full-length hCD81 of 90% (xt), 87% (dr), 73% (hCD9), 64% (hTSN32), 61% (hCD82), 60% (ce), and 58% (dm) (Figure 1B and Table 1). All chimeric hCD81 molecules carried a tandem hemagglutinin (HA) tag at their C-terminal cytoplasmic tail (Figure 1A,B). Chimeras had a similar size (233–242 aa) as hCD81 (236 aa), except hTSN32 (317 aa).

As the hCD81 LEL binds HCV E2, we first asked whether the hCD81 LEL was correctly folded and thus would bind HCV E2 when expressed in the context of the different tetraspanin backbones. To this end, we transduced CHO745 cells with each of the seven chimera constructs or with full-length hCD81. CHO745 cells transduced with an empty vector served as negative control (Ctrl). Since CHO745 cells have reduced glycosaminoglycan levels they show little background binding of HCV E2 glycoprotein. Four of the chimeras were expressed on the surface of CHO745 cells comparable to parental hCD81 (>80% positive cells). The hTSN32 and hCD82 chimeras were undetectable and the ce chimera showed reduced levels of 40% positive cells (Figure 1C,D). This smaller population of hCD81 LEL positive cells was reflected by a reduced mean fluorescence intensity (MFI) of 42,000 compared to 91,000 and higher for the other chimeras (Figure 1E). In sum, five of the seven hCD81 chimeras localized to the CHO745 cell surface and were used for further functional analysis.

**Figure 2:**
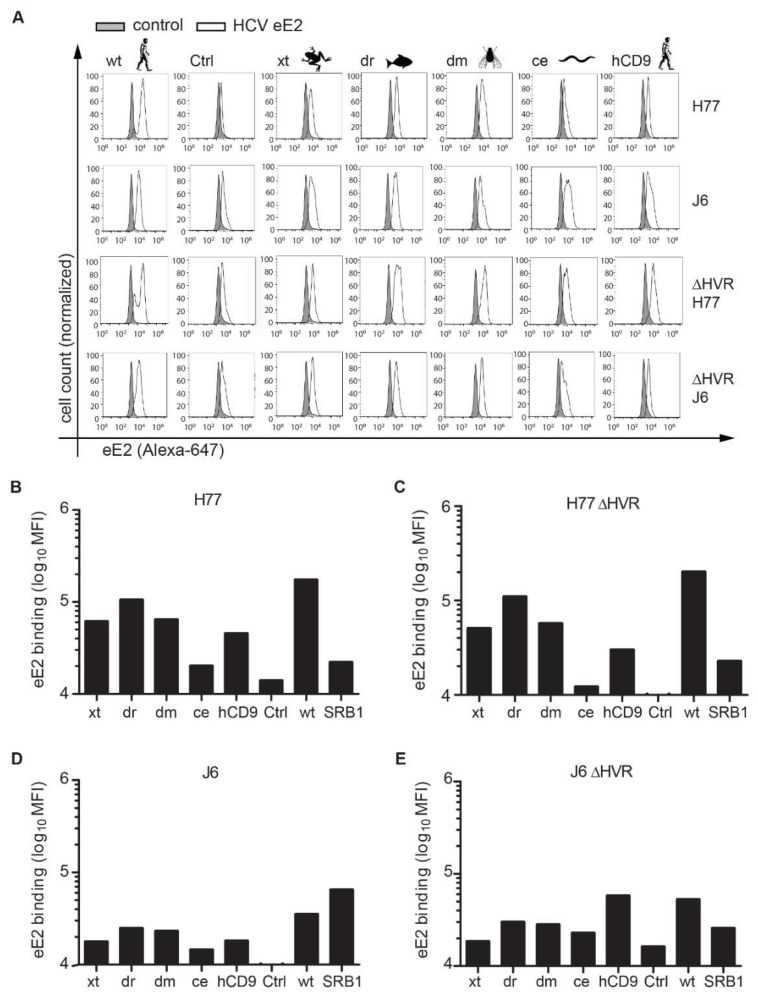
HCV E2 differentially binds to CHO745 cells expressing hCD81 backbone chimeras. (**A**) Binding of Alexa-647 labeled soluble E2 ectodomains (eE2) of the HCV genotypes 1a (strain H77) and 2a (strain J6) with and without the hypervariable region (HVR) to CHO745 cells expressing the indicated hCD81 chimera, full-length protein (wt) or empty vector control (Ctrl). Each histogram shows fluorescence intensities of untreated cells (grey) and cells after Alexa-647 eE2 binding (white); (**B**–**E**) Quantitation of eE2 binding to CHO745 cells expressed as MFI of Alexa-647. CHO745 cell expressing SR-BI served as additional positive control. Data show one representative experiment of three independent biological replicates.

We then labeled HCV E2 ectodomains (eE2) from genotype 1a (strain H77) and 2a (strain J6) with Alexa fluorophores and tested for their binding to the respective CHO745 cells (Figure 2A). CHO745 cells transduced with SR-BI served as additional positive control [41]. Both eE2 from H77 and J6 bound to cells expressing SR-BI (MFI 22,000 and 65,000, respectively), hCD81 (MFI 170,000 and 35,000, respectively) and the xt, dr, dm, and hCD9 chimeras (45,000–105,000 and 18,000–25,000, respectively) (Figure 2B,C). CHO 745 cells expressing the ce chimera bound eE2 with reduced efficiency (20,000 and 15,000, respectively) (Figure 2B,C). The reduced binding of eE2 to the ce chimera correlated with the reduced chimera cell surface expression level (Figure 1D). To enhance eE2 binding to the surface expressed hCD81 LEL, we performed the same binding assay with eE2 proteins deleted in their hypervariable region 1 (ΔHVR1). Deletion of this E2 domain is thought to increase CD81 binding and at the same time decrease SR-BI binding [11,51,52]. In line with these reports, the deletion of HVR1 from genotype 2a (J6) eE2 enhanced binding to the hCD81 chimeras. For instance, binding to the hCD9 chimera increased three-fold for ΔHVR1 J6 eE2 as compared to wildtype J6 eE2 (Figure 2B or Figure 2D). For genotype 1a (H77) ΔHVR1 eE2, hCD81 binding was not increased (Figure 2C or Figure 2E) suggesting that H77 eE2 binding to the hCD81 LEL is not shielded by the HVR1 region in this assay. As expected, ΔHVR1 eE2 from J6 strain bound to SR-BI expressing CHO745 cells less efficiently than full-length eE2 (Figure 2C or Figure 2E). In summary, all surface expressed hCD81 chimeras bound eE2 from HCV genotypes 1a and 2a to a degree that correlated with the number of chimera positive cells.

### 3.2. CD81-Tetraspanin Chimeras Are Expressed on the Surface of Human Hepatoma Cells

**Figure 3:**
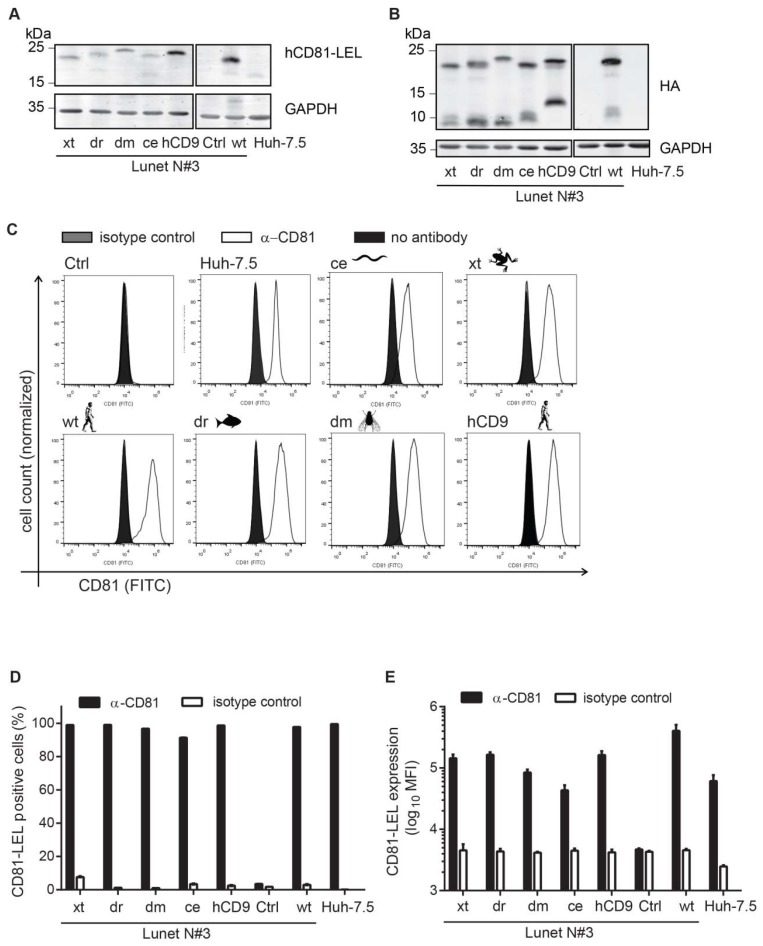
Generation and characterization of hCD81 chimera expressing human hepatoma cells. (**A**,**B**) Immunoblot for hCD81 LEL (**A**) and HA tag (**B**) in lysates from Lunet N#3 human hepatoma cells stably transduced with the indicated tetraspanin construct. GAPDH served as loading control and lysates from Huh-7.5 cells with endogenous hCD81 as positive control; (**C**–**E**) Cell surface staining of hCD81 on transduced Lunet N#3 cells as described in Figure 1C–E. Immunoblots and histograms are representative of at least three independent experiments and histograms show 30,000 cells per measurement. Mean + SD of three independent experiments shown.

To test whether the hCD81 chimeras function in HCV entry, we transduced hCD81-negative Lunet N#3 human hepatoma cells [39] with the chimeric constructs. All chimeras were detected in whole cell lysates by immunoblot at their predicted molecular weight (MW) using either anti-HA or anti-hCD81 LEL antibodies (Figure 3A,B). Lunet N#3 cells carrying an empty vector (Ctrl) or the full-length parental hCD81 construct served as negative and positive control, respectively. Huh-7.5 cells displayed lower levels of CD81 than the overexpressing cells. Endogenous CD81 in Huh-7.5 cells was 2.8 Da smaller than HA-tagged wt CD18 as expected (Figure 3A). For wt hCD81 and all chimeras we detected additional HA-positive bands at a MW lower than the predicted MW (Figure 3B). These truncated variants were previously observed [18]. Next, we determined the amount of hCD81 chimera surface expression using an antibody targeting the hCD81 LEL (clone JS-81). All five chimeras were expressed at the cell surface (>90% positive cells) (Figure 3C,D). Surface densities of xt, dr, hCD8 (MFI ~150,000), and dm (85,000) chimeras were slightly higher than endogenous hCD81 density in Huh-7.5 cells (61,000) but lower than overexpressed hCD81 (404,000) (Figure 3E). In sum, the five CD81 chimeras (xt, dr, ce, dm, and hCD9) traffic to the plasma membrane.

**Figure 4:**
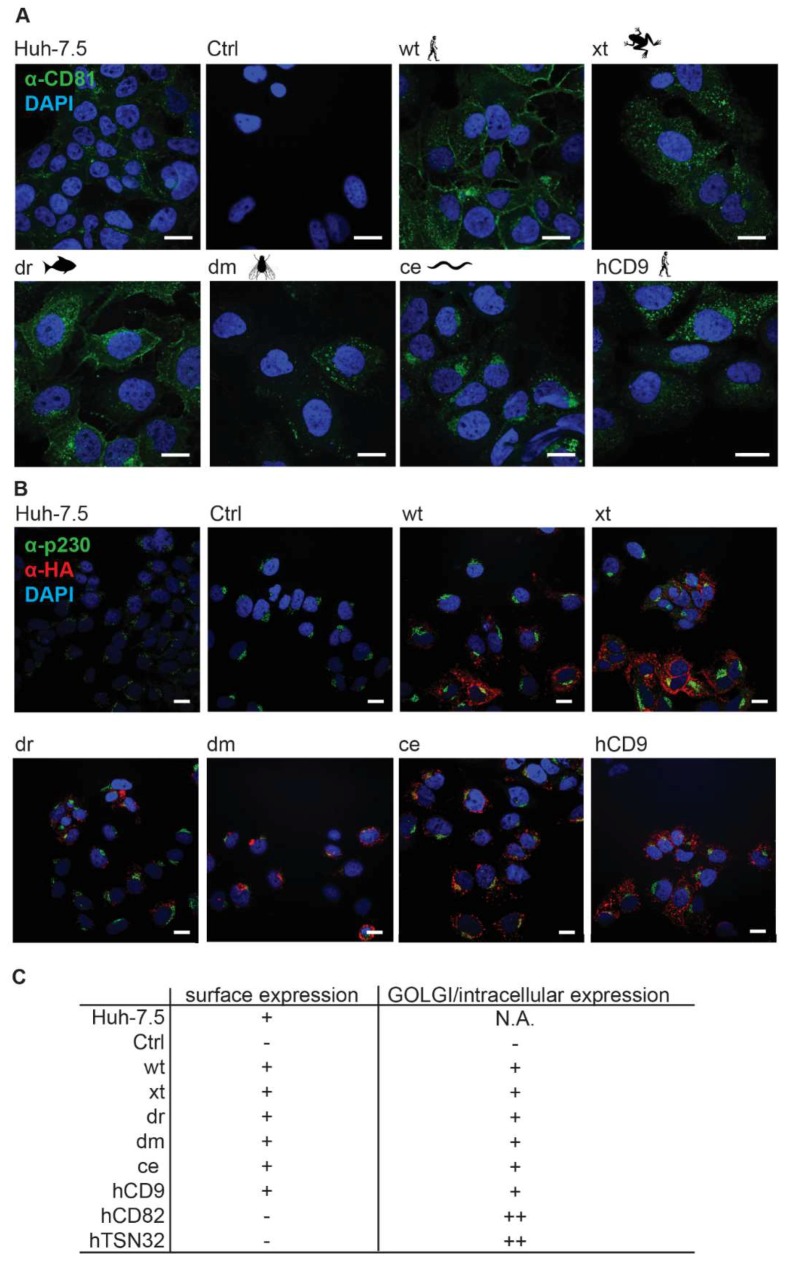
Subcellular distribution analysis of the five surface expressed hCD81 chimeras and full length hCD81 in Lunet N#3 and Huh-7.5 cells. (**A**) Confocal microscopy of hCD81 LEL stained Lunet N#3 cells expressing the indicated tetraspanin chimeras. Cells were permeabilized and stained with anti-hCD81 antibody (clone JS-81, green). Nuclei were stained with DAPI (blue). Huh-7.5 cells served as positive control, scale bars = 20 µm (**B**) Confocal microscopy of Lunet N#3 and Huh-7.5 cells stained with anti-HA antibody (red), anti-p230 antibody (green) as Golgi marker and DAPI (blue). Representative images; scale bars = 20 µm. (**C**) Semi-quantitative analysis of hCD81 chimera subcellular localization. +, expression observed; −, expression not observed; N.A., data not available.

We further assessed the subcellular localization of the seven chimeras in Lunet N#3 cells by confocal microscopy (Figure 4B). Parental hCD81 localized to the cell surface and to intracellular compartments including the Golgi apparatus. A similar staining pattern was observed for the five chimeras (hCD9, xt, dr, dm, and ce), however with somewhat less surface staining and more or less dispersed intracellular staining. The hCD82 and hTSN32 chimeras, which we excluded from further analysis due to their lacking surface expression, indeed exclusively localized to the Golgi (Figure S1A–D). Thus, immunofluorescence microscopy confirms surface targeting of hCD9, xt, dr, dm, and ce chimeras.

### 3.3. The hCD81 Backbone Influences HCV Pseudoparticle Infectivity

**Figure 5:**
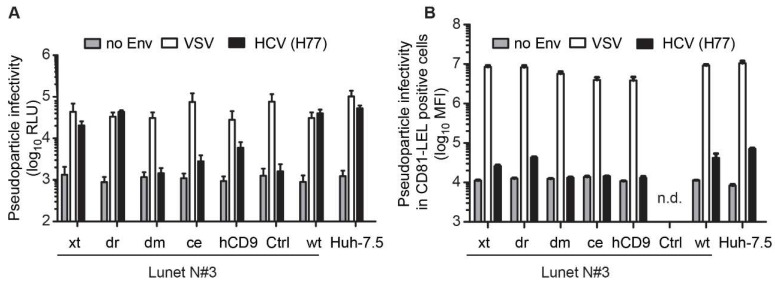
The hCD81 backbone influences HCV pseudoparticle infectivity. (**A**) Chimera expressing Lunet N#3 cells and Huh-7.5 cells were transduced with lentiviral pseudoparticles encoding Firefly luciferase and displaying the glycoproteins of HCV (strain H77) or VSV. Pseudoparticles without glycoproteins (no Env) served as negative control. Pseudoparticle infectivity was measured at 72 hpt by quantitation of luciferase activity; (**B**) Infectivity of GFP encoding lentiviral pseudoparticles displaying HCV (H77) glycoproteins, VSV glycoproteins, or no glycoproteins in chimera expressing Lunet N#3 cells and Huh-7.5 cells. At 72 hpt cells were counterstained with anti-hCD81 antibody, and infectivity of lentiviral GFP pseudoparticles in the hCD81 LEL positive subpopulations quantified. n.d., not detected, i.e., CD81 positive cells below 1000 counts. All experiments were performed at least thrice and are represented as mean + SD of three independent biological replicates (**A**) or technical triplicates (**B**).

To explore whether the hCD81 backbone is a critical determinant for HCV entry, we next infected the hCD81 chimera expressing Lunet N#3 cells with lentiviral pseudoparticles displaying the HCV genotype 1a (H77) glycoproteins on their surface. Pseudotypes with VSV glycoprotein (VSV G) served as positive control and pseudotypes without surface glycoproteins as negative control (no Env). The lentiviruses encoded Firefly luciferase for quantitation of infection rates. All cell lines conferred VSV G mediated pseudotype entry with similar efficiency. However, they displayed clear differences in HCV glycoprotein mediated entry (Figure 5A). Xt and dr chimeras supported HCV pseudoparticle entry comparable to parental hCD81. In contrast, ce and hCD9 chimeras conferred tenfold lower entry levels than hCD81. Dm chimeras completely failed to support HCV pseudoparticle entry (Figure 5A). After normalizing HCV entry to VSV G mediated entry, it became apparent that ce chimera expressing cells displayed strongly reduced HCV entry efficiency, similar to the dm cells (Figure S2A). Using pseudoparticles encoding GFP and analyzing infectivity exclusively in hCD81 LEL positive cells, we confirmed these observations (Figure 5B and Figure S2B,C). Taken together, we observed a gradual decrease in HCV pseudoparticle susceptibility that was directly linked to the degree of homology of the chimera to hCD81.

### 3.4. Entry of HCV Cell Culture Virus Depends on the hCD81 Backbone

**Figure 6:**
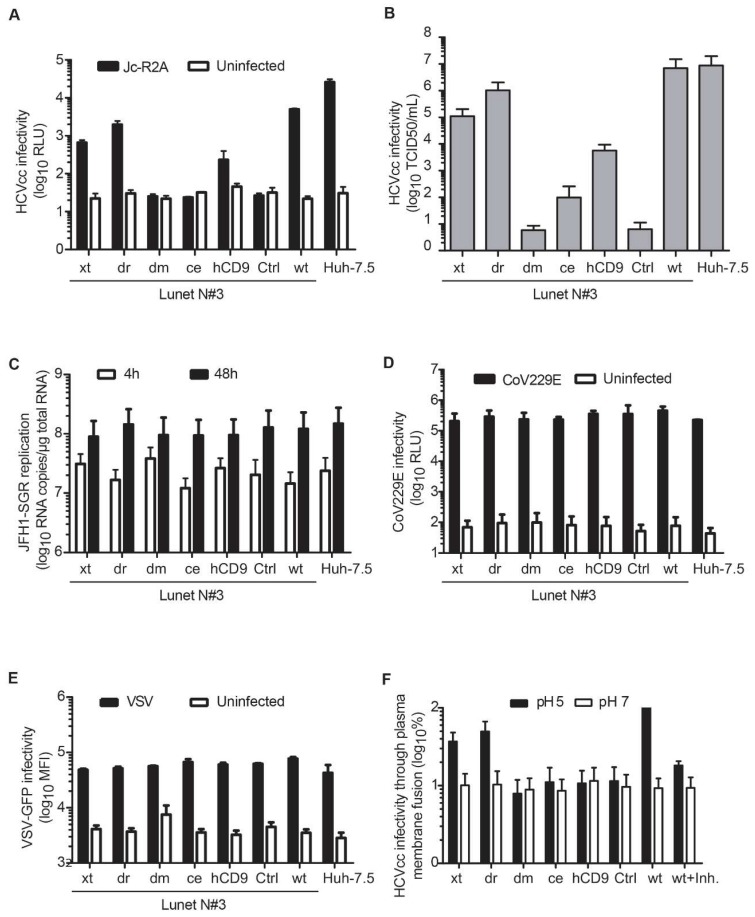
The hCD81 backbone determines HCV infectivity, but not human coronavirus and VSV infectivity. (**A**) Infectivity of a luciferase reporter HCV strain (Jc-R2A) in Lunet N#3 cells expressing hCD81 chimeras and in Huh-7.5 cells. Cells were infected for 72 h at an MOI of 0.5 and infectivity quantified as luciferase activity in cell lysates; (**B**) Infectivity of non-reporter full-length HCV (Jc1) in hCD81 chimera expressing cells. Viral titers at 72 hpi were determined by limiting dilution on Huh-7.5 cells and immunocytochemical staining against the viral NS3 protein; (**C**) Replication assay using an HCV subgenome (JFH-1). Cell lines were electroporated with JFH-1 subgenomic RNA and intracellular viral RNA was quantified by real time PCR at the indicated timepoint; (**D**) Human coronavirus (strain 229E) infectivity determined at 48 hpi using Renilla luciferase reporter virus; (**E**) VSV infectivity determined at 16 hpi using GFP reporter virus; (**F**) HCV (JcR2A) infectivity through artificially induced plasma membrane fusion. Virus was bound in the presence of a V-type ATPase inhibitor and fusion induced at the plasma membrane by a low pH wash. The fusion inhibitor flunarizine (Inh.) served as control. Data shown as mean + SD (SEM in (**F**)) of three to five independent biological replicates each performed in technical triplicates.

**Figure 7:**
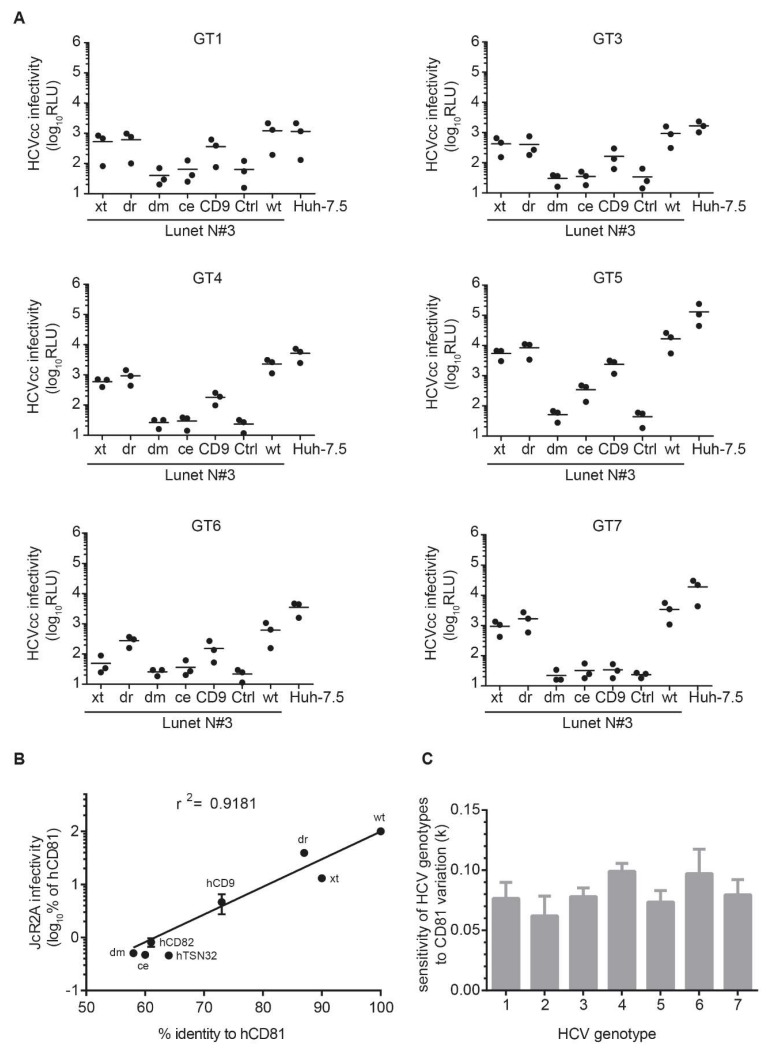
HCV genotypes similarly depend on the hCD81 backbone. (**A**) Lunet N#3 cells expressing the hCD81 chimeras and Huh-7.5 cells were infected with intergenotypic chimeric viruses encoding Renilla luciferase reporter. At 72 hpi, cell lysates were analyzed for luciferase activity as a measure of HCV infectivity. Graphs show three independent biological replicates (mean of technical triplicates each); (**B**) Influence of CD81 variation on HCV susceptibility of Lunet N#3 cells. The HCV genotype 2a susceptibility (normalized to hCD81 expressing Lunet N#3 cells) is plotted on the y axis and the identity of the respective CD81 chimera to hCD81 on the x axis. Exponential growth regression analysis with all negative values excluded from analysis; (**C**) Sensitivity of HCV genotypes to CD81 backbone variation plotted as rate constant (k) of the exponential growth regression analysis for HCV genotype 1–7 intergenotypic chimeras. Statistical significance in (**C**) tested by ANOVA with no significant differences observed.

HCV circulates as lipoviroparticle in the blood and the association with serum lipoproteins can influence virus entry events. To test if the hCD81 backbone affected HCV lipoviroparticle entry, we next infected backbone chimera cells with cell culture derived HCV (HCVcc). Using a Jc1-based Renilla luciferase reporter virus (JcR2A), we found that the dr chimera rendered Lunet N#3 cells similarly susceptible (2000 RLU) as full-length hCD81 (5000 RLU). The xt and hCD9 chimeras less efficiently supported HCVcc infection (700 RLU and 200 RLU, respectively) (Figure 6A). Dm and ce chimera expressing cells were refractory to HCVcc infection. These results were confirmed with a non-reporter genotype 2a HCVcc strain (Jc1) (Figure 6B). To assess if the HCV entry efficiency reflects hCD81-LEL chimera surface expression levels, we performed regression analysis of infectivity and hCD81-LEL surface staining MFI. The best fit was achieved with a linear regression (r^2^ = 0.85, Figure S3). Dr and ce chimeras displayed a strong correlation between surface expression levels and HCV susceptibility. In contrast, hCD9, xt and dm chimeras deviated from the regression curve and conferred lower HCV susceptibility than predicted according to their surface expression levels. A stronger correlation was observed when plotting HCV infectivity against hCD81 sequence homology (r^2^ = 0.92, Figure 7B). All tested proteins in particular hCD9 and dm chimeras displayed a positive correlation of homology and infectivity. In sum, HCVcc infection largely reflected the observed HCV pseudoparticle phenotypes in that only backbones closely related to hCD81 confer susceptibility to HCV.

Since HCVcc assays score infectivity late in the virus life cycle and therefore describe the sum of virus entry and replication, we next sought to exclude that the tested tetraspanin chimeras would influence HCV RNA genome replication. To this end, we transfected chimera expressing Lunet N#3 cells with subgenomic genotype 2a HCV RNA (JFH-1) and quantified intracellular RNA levels at 4 h and 48 h post transfection. HCV genome RNA copy numbers were comparable in all tested cell lines irrespective of the presence or absence of hCD81 chimeras (Figure 6C). Thus, the different usage of hCD81 chimeras by HCVcc depended on the ability of the chimeras to promote virus entry and not viral genome replication.

CD81 has previously been reported to have subtle effects on cell proliferation [53] and to be important for membrane microdomain formation [54,55]. To exclude effects of the hCD81 chimeras on cell proliferation or gross membrane organization, we assessed susceptibility to hCD81-independent enveloped viruses. Human coronavirus 229E (CoV) and vesicular stomatitis virus (VSV) infected hCD81 or tetraspanin chimera expressing Lunet N#3 cells with equal efficiencies (Figure 6D,E). Thus, tetraspanin chimera expression in Lunet N#3 cells did not affect generic cellular functions required for virus infection.

HCV entry is a multistep process involving receptor binding, endocytosis, membrane fusion and uncoating. To investigate, which post-binding function was impaired in the dm and ce chimeras, we bypassed the endosomal uptake route of HCV by blocking the V-type ATPase and artificially inducing virus particle fusion at the plasma membrane through low pH wash. Again, we observed a correlation between backbone sequence homology and cell susceptibility to HCV. While dr and xt chimeras supported HCV entry, the more distantly related dm and ce chimeras failed to support HCV entry. An inhibitor of HCV fusion, flunarizine [56], expectedly reduced fusion five-fold (Figure 6F). As plasma membrane fusion is rather inefficient, we failed to observe the intermediate phenotype of the hCD9 chimera. Taken together, we show that distantly related tetraspanin backbones, in particular the dm backbone, fail to support post-binding and endocytosis-independent HCV entry steps such as lateral membrane translocation, fusion and uncoating.

### 3.5. The Seven HCV Genotypes Depend Similarly on the hCD81 Backbone

Lastly, we asked whether all HCV genotypes had the same requirements for the CD81 backbone. Seven genotypes for HCV are reported and these differ strongly in the amino acid sequence of their structural proteins (up to 40% divergence) leading to differential usage of the entry factors OCLN and CLDN [57,58,59]. Therefore, we tested a panel of intergenotypic reporter viruses displaying structural proteins of the seven major HCV genotypes. While genotype one through five chimeras fully reflected the genotype 2a HCVcc phenotype in efficient usage of dr and xt and intermediate usage of hCD9 backbone domains, genotype six and seven chimeric viruses failed to use the xt and hCD9 backbones, respectively, for infection (Figure 7A).

To determine a possible correlation of tetraspanin sequence identity and HCV susceptibility, we performed regression analyses. Using exponential growth regression, we observed a moderately good correlation (r^2^ = 0.92) for genotype 2a HCV (Figure 7B). Similar correlations were observed for genotypes 1 and 3–7. The parameters of the exponential growth regression analyses (rate constant k of the exponential regression curve) for each of the seven genotypes were statistically indistinguishable (Figure 7C). This suggests that, despite small differences in backbone requirements, the overall dependency on CD81 backbone domains is comparable between HCV genotypes.

### 3.6. The hCD81 Glutamate Residue 219 Contributes to HCV Susceptibility

**Figure 8:**
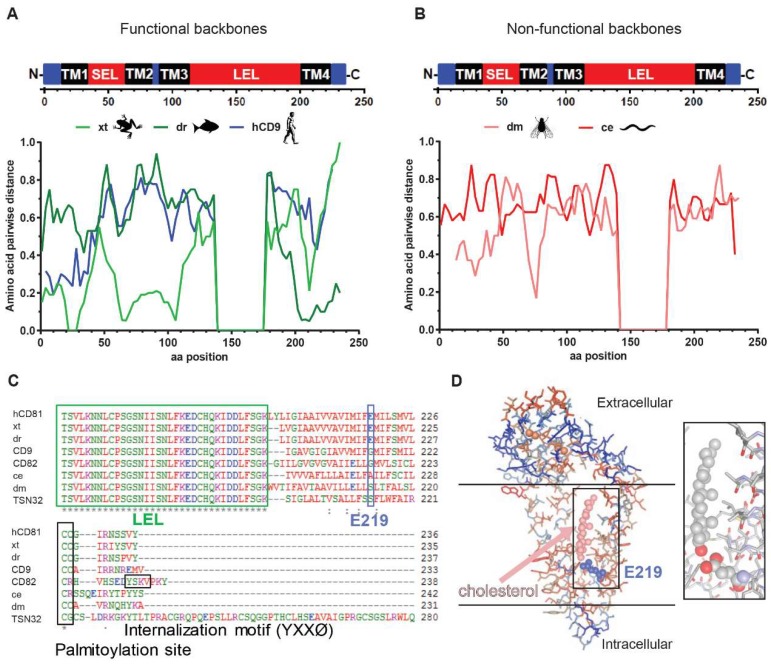

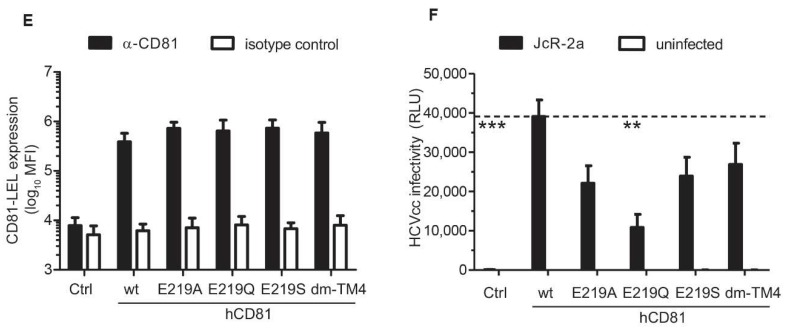
Transmembrane domain four of dmTSP90F and cdTSP9 critically differ from hCD81. (**A**,**B**) Scheme of the structural domains of hCD81 and amino acid sequence divergence scan of hCD81 chimeras with HCV entry supporting (**A**) or resistant (**B**) backbones; (**C**) Amino acid sequence alignment of the transmembrane domain four (TM4) and the N-terminus of the tetraspanin chimeras used in this study. The cholesterol binding residue E219 is highlighted in light blue. A conserved palmitoylation site and an internalization motif are highlighted in black; (**D**) Structure of full length hCD81 with bound cholesterol (light red, ball and stick) and the E219 residue (blue, ball and stick). The boundaries of the plasma membrane are indicated by horizontal lines. (PDB: 5TCX) [19]. CD81 amino acids are colored according to the side chain hydrophobicity (polar: blue, nonpolar: red). Disulfide bonds in LEL highlighted in orange. The insert shows the zoom into the E219–cholesterol interface formed by hydrogen bonds between the E219 carboxyl group and the cholesterol hydroxyl group (oxygen atoms labelled in red, nitrogen in blue). (**E**) Cell surface staining of hCD81 on TM4 mutant transduced Lunet N#3 cells as described in Figure 1D. (**F**) Infectivity of luciferase reporter HCV strain (Jc-R2A) in hCD81 TM4 mutant expressing Lunet N#3 cells as described in Figure 6A. Data shown as mean + SEM of four independent biological replicates each performed in technical triplicates. ** *p* ≤ 0.01, *** *p* ≤ 0.001.

To understand which tetraspanin backbone domains caused the observed deficiency in HCV receptor function, we determined the sequence divergence of all tested chimeras from hCD81. The functional chimeras (xt and dr) differed in their homology profiles from the non-functional chimeras. While, in most backbone domains, including the transmembrane domains (TM) 1–3 and the cytoplasmic tail regions, at least one of the chimeras displayed a strong divergence (Figure 8A), high sequence conservation was observed in transmembrane domain four (TM4). The non-functional chimeras, dm and ce, showed high sequence divergence throughout most backbone domains (Figure 8B). Strikingly, the degree of sequence homology in TM4 correlated with the observed HCV susceptibility phenotype with the following order of increasing homology and infectivity: dm < ce < hCD9 < xt < dr.

We further investigated which residues in TM4 and the rest of the backbone of hCD81 may result in the observed phenotype. Juxtamembrane cysteine palmitoylation sites were conserved in all tested tetraspanins. The C-terminus of hCD82 additionally carries a YxxØ internalization motif, which may cause its intracellular retention (Figure 8C). A glutamate residue (E219) in TM4 of hCD81, which forms hydrogen bonds with cholesterol, lacks in the hCD9 chimera with intermediate receptor function (Figure 8C,D and Table S1) [19]. Hydrophobicity analysis confirmed that glutamate at position 18 of TM4 is indeed missing in the hCD9 chimera and located at position 13 in ce and position 15 in dm (Figure S4A–D). Notably, hCD81 residues predicted to be important for CLDN1 interaction were fully conserved in the here tested tetraspanin chimeras (Table S2) [21].

To test for the role of E219 in HCV infection, we generated cell lines expressing E219Q, E219A and E219S point mutations, the latter two reflecting the residues in ce and dm chimeras. Additionally, we included a hCD81 chimera carrying the dm TM4 (hCD81 dm-TM4). Surface expression of all hCD81 variants was comparable to hCD81 as demonstrated by antibody staining and flow cytometry (Figure 8E). HCVcc infectivity, however, was impaired two- to four-fold in all four chimeras with the strongest phenotype displayed by the E291Q mutation (Figure 8F). All cell lines supported HCV RNA genome replication with similar efficiencies confirming an entry phenotype (Figure S4E). The hCD81 dm-TM4 chimera cell line was impaired in HCV infection but not as strongly as the dm cell line, suggesting that TM4 is a HCV susceptibility determinant, but additional backbone domains are required. Taken together, this work shows the critical post-binding role of the hCD81 backbone, including residue E219, in HCV infection and further underlines the multiple functions of hCD81 in HCV entry.

## 4. Discussion

Virus receptors mediate virus attachment to the cell surface and coordinate productive uptake of the viral genome, which is essential for virus propagation [1]. The HCV E2 glycoprotein binds to the LEL of the hCD81 receptor and this interaction is critical for HCV infection [8,34]. Contributions of other hCD81 domains to HCV uptake had only partially been addressed previously [24,60,61]. Here, we demonstrate that hCD81 backbone domains are important for HCV uptake and confirm that the hCD81 backbone domains are more resistant to sequence alterations than the LEL [24,27,62]. We demonstrate that backbones with moderate sequence similarity to hCD81 (at least 64% amino acid identity) can fully substitute the hCD81 function in HCV entry. More distantly related backbones partially support HCV entry (hCD9 with 45% amino acid identity) or fail to support HCV entry (dm tetraspanin with 27%, ce tetraspanin with 25% amino acid identity) (Table 1).

The dm CD81 orthologue backbone, with an hCD81 amino acid sequence similarity of 27%, rendered Lunet N#3 cells resistant to HCV despite proper folding and plasma membrane expression of the dm chimera. Specifically, dm chimera cell surface expression levels were as high as for endogenous hCD81, which others and we show is sufficient to confer maximum susceptibility to HCV (Figure 3E and Figure 5A) [63]. The LEL of hCD81 seemed to be correctly folded in the context of the dm tetraspanin backbone, as soluble HCV E2 glycoprotein and a conformation sensitive hCD81 antibody could bind to CHO cells expressing the chimera. Thus, the hCD81 LEL displays a fairly high tolerance for backbone domain exchanges, which is in line with a previous report on hCD81-hCD82 chimeras (26% sequence identity) [27]. In accordance with the hCD82 study, we observe resistance of dm chimera expressing cells to HCV entry. Thus, the hCD81 backbone, in addition to the hCD81 LEL, is a critical HCV susceptibility determinant.

A possible function of the hCD81 backbone may be the interaction with proteins or lipids essential for HCV uptake. Here, we demonstrate a strong correlation between sequence homology in transmembrane domain four of hCD81 and HCV entry factor function, which is in line with previous findings of a role of TM3 and TM4 in HCV infection [27]. In particular, E219, which is important for cholesterol binding, was lacking in the less functional hCD9 backbone chimera. Cholesterol is required for efficient HCV entry [11,64,65,66,67] and mutations of E219 reduce hCD81 cholesterol binding [19]. Whether direct cholesterol coordination by hCD81 is required for HCV infection was previously unknown. We show that E219 point mutations in hCD81 reduce HCV infectivity up to four-fold. This suggests that hCD81 coordination of cholesterol directly impacts HCV infection. Since we only observed a four-fold reduction of susceptibility to HCV upon E219 mutation, additional residues in the hCD81 backbone play an important role. This is in line with our observation that dm and ce chimeras, which express glutamate in TM4, are strongly impaired in HCV receptor function. Of note, in particular for the ce chimera, we cannot rule out that reduced chimera surface expression levels cause the impaired HCV receptor function. A contributing but not exclusive role of E219 is further supported by the observed 10-fold reduction in HCV infection for the CD9 chimera, which lacks E219. Thus, E219 is one of several critical residues in the CD81 backbone required to confer full susceptibility to HCV.

Two tested chimeras (hCD82 and hTSN32) were retained in the Golgi. Of note, hCD82 and hTSN32 were among the most distantly related tetraspanins (26% and 15% amino acid identity) in our panel of seven tetraspanins (Table 1). This may suggest that the hCD81 LEL can only be inserted into tetraspanins with a minimum threshold sequence identity and that too distantly related tetraspanins do not tolerate the LEL exchange. Previously, a hCD82 backbone chimera was reported to show low but detectable levels of surface expression in CHO and Huh-7w7 cells [27]. Possibly, the combination of the C-terminal tandem HA tag and the hCD82-hCD81 fusion might lead to a more pronounced misfolding or trafficking deficiency observed in this study. The intracellular localization of the hCD82 chimera observed in our study is however in line with an N-terminal internalization motif in hCD82.

A third function of the hCD81 backbone in addition to cholesterol coordination and surface targeting may be the interaction with host proteins, which act as HCV entry co-factors. Human CD81 forms a complex with the entry factors SR-BI and CLDN1 [21,22], the HCV entry co-factors HRas and SRFBP1 [22,23,68] and the entry restricting factor IGSF8 [27]. Except for IGSF8, hCD81 domains mediating the interaction with these co-factors remain elusive. It is likely that entry co-factors, similar to IGSF8, rely on hCD81 backbone domains for complex formation. In particular HRas and SRFBP1, which localize to the cytosolic membrane leaflet, can only associate with the HCV receptor complex via backbone domains of hCD81 [22,23]. Interaction with the HCV entry factor CLDN1 (Table S2) [21], however, is largely mediated by the LEL and is thus likely not impaired in the here described chimeras.

While our study points towards an important contribution of non-LEL regions of CD81 during HCV entry, the study has limitations. These include the use of non-polarized hepatoma cells (Huh-7 derived clones). HCV entry in polarized cells seems to strongly rely on CD81 interactions with host proteins leading to the activation of the GTPases Rac, Rho and Cdc42 and subsequent actin dependent transport of the HCV–CD81 complex to sites of entry [14,22,69]. As interactions with cytosolic proteins likely rely on non-LEL regions of CD81, it is conceivable that the effects observed in Huh-7 derived cells in this study, may be even more pronounced in polarized cells such as HepG2 cells. A second caveat may be direct effects of chimera generation on the observed phenotypes. In particular, LEL and non-LEL regions of chimeras may interact differently from homologous LEL and non-LEL regions and this in turn may influence CD81 chimera association with additional proteins. Thus, chimera tetraspanin networks may be altered and not fully reflect those of the full-length proteins in the corresponding species environment. Third, non-LEL regions from a particular species are expressed in a human environment in our study, which can of course alter protein–protein interactions occurring in the natural environment. While the full set of CD81 network proteins in human liver cells is not yet described, we did control for proper folding of the hCD81 LEL in the context of all non-LEL regions from other species. HCV E2 binding as well as binding of a conformation sensitive LEL antibody strongly suggest, that the LEL is correctly folded to capture HCV virus particles. Future studies will reveal, which HCV entry relevant interactions of CD81 are mediated by the non-LEL regions. Major alterations of LEL–non-LEL interactions can be excluded for the single point mutant at position E219, which may indeed explain the relatively small fold change of infectivity for this mutant. Clearly, our data suggest that E219 is one of several residues in the CD81 non-LEL region important for HCV infection.

The endogenous cellular function of CD81 has been characterized in detail in B and T cells, where it provides a costimulatory signal for the B and T cell receptor complex, respectively. This function critically relies on CD81 interactions with additional proteins of the B and T cell receptor complex (reviewed in [54]). In hepatoma cells, the endogenous function of CD81 is less well defined, although it has been implicated in cell migration and proliferation upon tissue wounding [53,70]. In this study, we exclusively analyzed the role of CD81 in the context of HCV infection. We excluded that the observed effects on HCV infection were due to a general impairment in cell growth or function by performing VSV and CoV infection assays as well as HCV RNA replication assays. In none of the latter experiments, the CD81 chimera expressing or empty vector expressing cells were impaired, suggesting that CD81 chimeras directly affected HCV infection. Future work will reveal if any of the endogenous roles of CD81 in liver cells is impaired by non-LEL region exchanges.

Similar to our observations with HCV, tetraspanin backbone domains are known to impact papillomavirus infection. Papillomavirus types 16, 18, and 31 require the tetraspanin CD151 during cell entry [71,72], and the CD151 C-terminal domain and juxtamembrane palmitoylation sites are critical for infection [72]. Both domains mediate PPIs; the C-terminus coordinates CD151-integrin complexes, and the palmitoylation guides lateral interactions with other tetraspanins thereby positioning CD151 in tetraspanin enriched membrane microdomains. CD81 palmitoylation also leads to membrane microdomain localization [18,27,73], but the role of CD81 palmitoylation in HCV entry is disputed [27,74]. In this study, all tested chimeras displayed palmitoylation sites, demonstrating that the tetraspanin backbone can impact virus entry through additional residues such as the cholesterol coordinating E219. Since several other viruses including chikungunya virus, HIV-1 and cytomegalovirus use tetraspanins during entry and spread [75,76,77,78], our work may inform about critical tetraspanin domains and residues in the context of other viral infections.

In addition to full-length proteins, we detected truncated variants of hCD81 and the chimeric tetraspanins using anti-HA antibodies. A previous study using tagged hCD81 reported truncations of similar molecular weight [18]. Whether these truncated forms resulted from proteolytic cleavage is unclear. The truncated variants were insensitive to hCD81 antibodies. Therefore, it is unknown, whether endogenous hCD81 is similarly processed. If so, testing for a possible cellular function of the short hCD81 variant would be of high interest as truncated scavenging variants of receptors have been reported before [79].

Serum derived and cell culture derived HCV particles are associated with serum lipoproteins [80,81,82,83,84,85] and this association determines entry steps such as the attachment to low density lipoprotein receptor [13,86,87]. Using lentiviral pseudotype viruses, which are devoid of serum lipoproteins, we could show that the hCD81 backbone acts independently of the virion association with lipoproteins. This suggests that HCV-relevant and backbone mediated functions of hCD81 are either steady state functions or E2 glycoprotein primed, but not lipoprotein primed functions.

HCV genotypes differ greatly in their E1/E2 glycoproteins and are differentially distributed geographically [88]. We found that hCD81 backbone requirements were mostly similar for all genotypes. However, genotype 2 tolerated slightly more changes in the backbone than the remaining six genotypes. This is in line with the strong cell culture adaptation of genotype 2 HCV strains [89].

In conclusion, our work provides further evidence that hCD81 plays a role in HCV particle binding and signaling for productive HCV entry. While the LEL of hCD81 mediates virus binding, the hCD81 backbone domains are critical to signal for productive entry. These results have implications for the assessment of human genetic CD81 variants and their associated susceptibility to HCV.

## Figures and Tables

**Table 1:** Amino acid (aa) sequence identities of hCD81 paralogs, orthologs and chimeras used in this study.

Protein	Length	Identity with hCD81 (aa)	hCD81-LEL Chimera (w/o HA-Tag)	Length	Identity with hCD81 (aa)
hCD81	236	100%	hCD81	236	100%
xtCD81	237	72%	xt	235	90%
drCD81	236	64%	dr	237	87%
hCD9	228	45%	h	233	73%
dmTSP96F	268	27%	dm	231	58%
hCD82	267	26%	hCD82	238	61%
ceTSP9	233	25%	ce	242	60%
hTSN32	320	15%	hTSN32	317	64%

## Supplementary Materials

The following are available online at http://www.mdpi.com/1999-4915/10/4/207/s1, Figure S1: Subcellular localization of two intracellular retained CD81 chimeras in Lunet N#3 cells, Figure S2: The hCD81 backbone influences HCV pseudoparticle infectivity, Figure S3: Correlation analysis of HCV infectivity and hCD81-LEL surface expression in Lunet N#3 cells, Figure S4: Transmembrane domain four residues in dmTSP90F and cdTSP9 critically differ from hCD81, Table S1: Residues in hCD81 and dmTSP96F backbones with critical functions, Table S2: Critical residues in the hCD81 LEL and their function.
