# Supplementary material for: CD81 Receptor Regions outside the Large Extracellular Loop Determine Hepatitis C Virus Entry into Hepatoma Cells
Source: Viruses. 2018 Apr 20;10(4):207. doi: 10.3390/v10040207 (PMC5923501; doi:10.3390/v10040207)
Supplement: Supplementary file 1 [file viruses-10-00207-s001.zip › CD81 backbone Supplement/CD81 backbone Supplemental Figures&Legends.pdf]

SUPPLEMENTAL FIGURES AND FIGURE LEDGENDS

S1

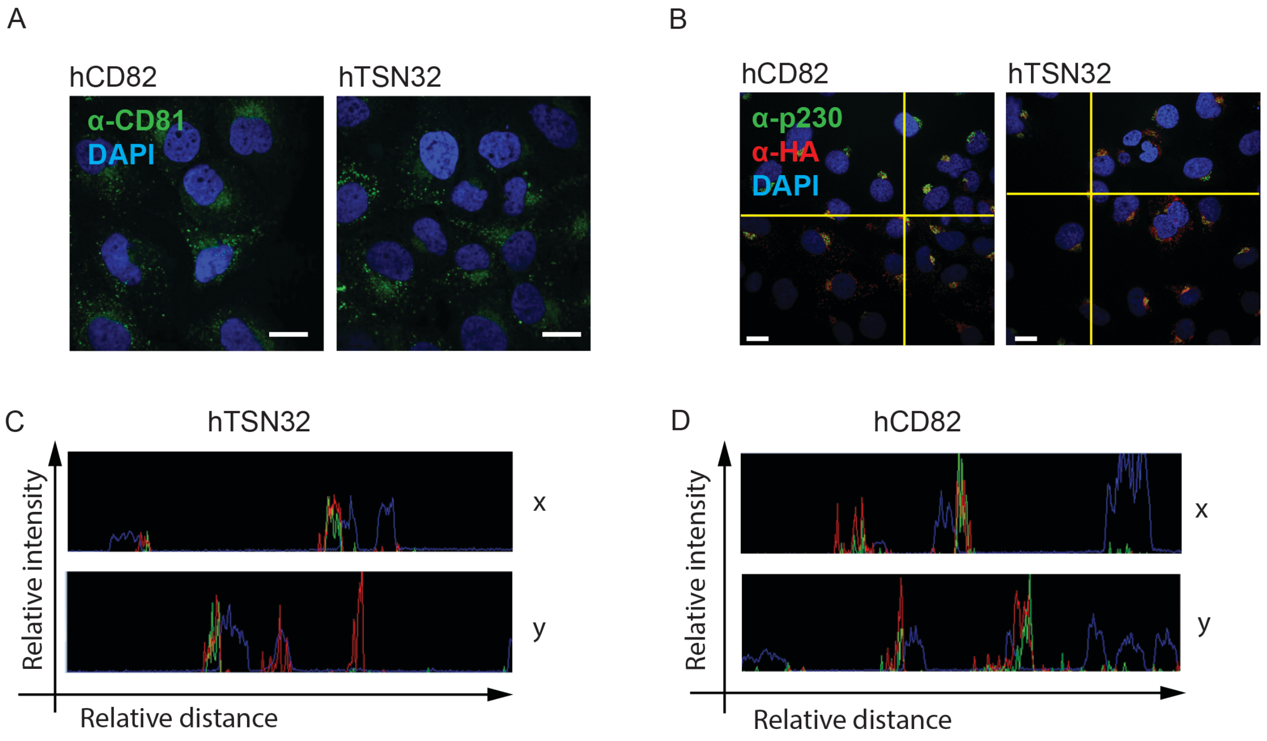

Fig. S1. – Subcellular localization of two intracellularly retained CD81 chimeras in Lunet N#3 cells. (A) Confocal microscopy of Lunet N#3 cells expressing the indicated tetraspanin chimeras as in Fig. 4. Permeabilized cells stained with anti-hCD81 antibody (green). (A) or with anti-HA antibody (red) and anti-p230 antibody (green) as Golgi marker (B). Nuclei were stained with DAPI (blue). (C and D) Intensity distribution analysis of the Golgi marker p230 and the anti-HA signal in hTSN32 and hCD82 expressing Lunet N#3 cells. The analyzed cross-sections are indicated in B as yellow lines. Representative images; scale bars 20 μm.

S2

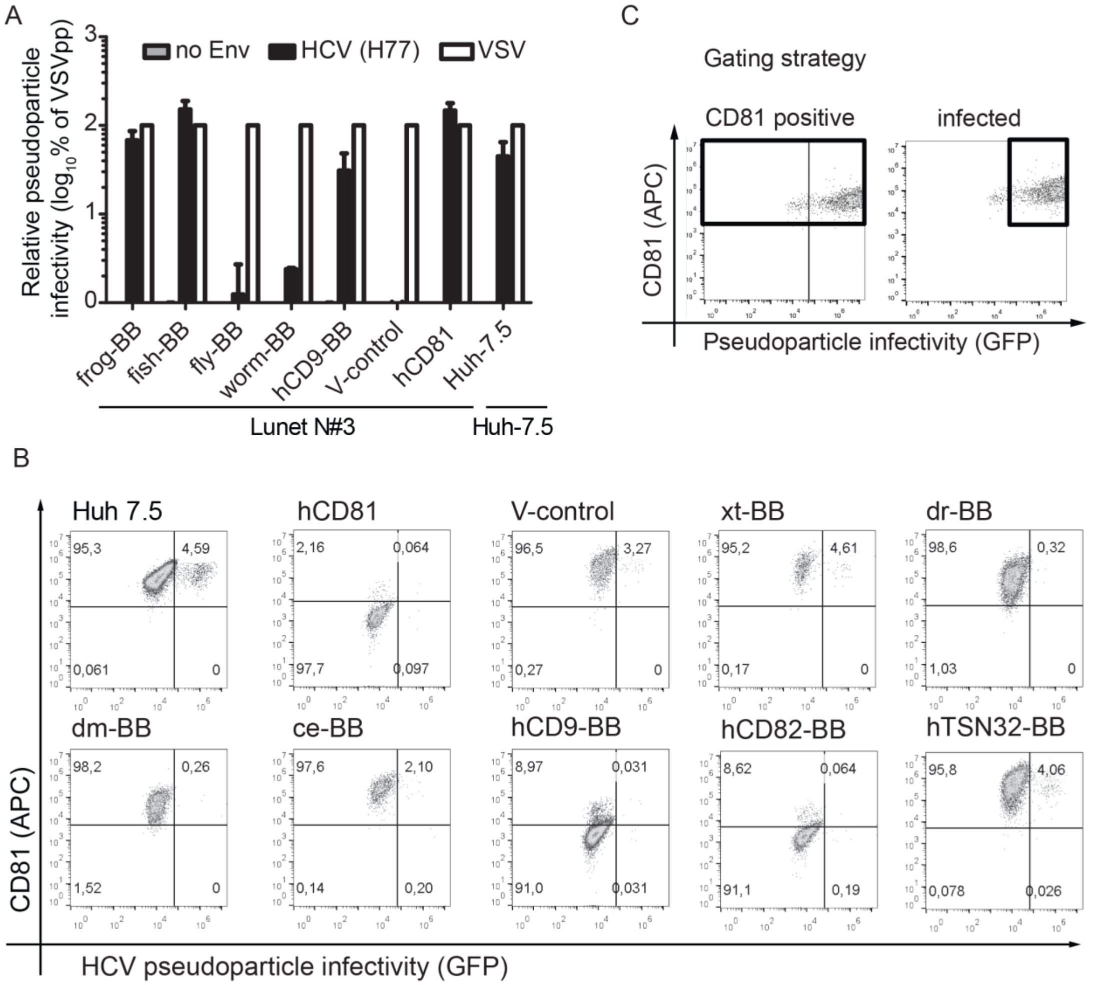

Fig. S2. – The hCD81 backbone influences HCV pseudoparticle infectivity. (A) Relative luciferase pseudoparticle infectivity after background subtraction (no Env) and normalization to VSV pseudoparticles. Infectivity of lentiviral pseudoparticles encoding Firefly luciferase and displaying the glycoproteins of HCV (strain H77), VSV or no glycoproteins (noEnv) assessed in chimera expressing Lunet N#3 cells and Huh-7.5 cells as described in Fig. 5A. (B) Infectivity of GFP encoding lentiviral pseudoparticles displaying HCV (H77) glycoproteins in chimera expressing Lunet N#3 cells and Huh-7.5 cells. At 72 hpt cells were counterstained with anti-hCD81 antibody. (C) Gating strategy to determine GFP pseudoparticle infectivity only in hCD81 LEL positive cells. n.d.: not detected. All experiments were performed at least thrice and are represented as mean + SD of three independent biological replicates. Flow cytometric dot plots are representative of three experiments and show at least 5,000 cells.

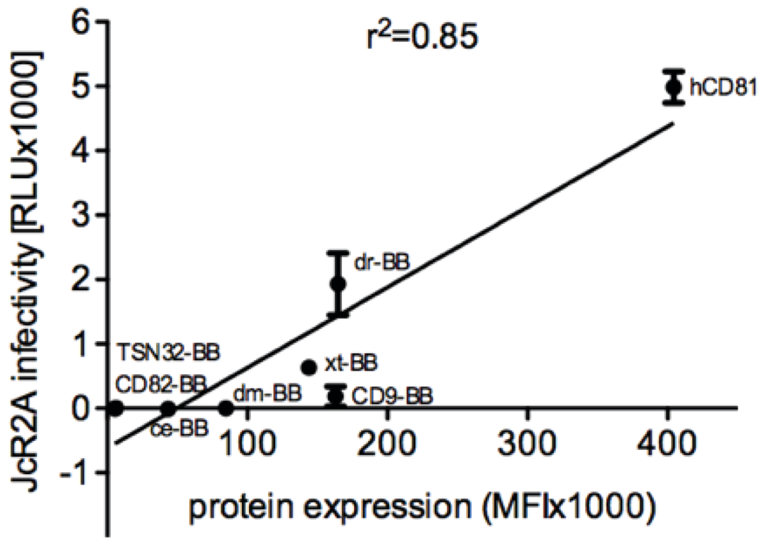

Fig. S3. – Correlation analysis of HCV infectivity and hCD81-LEL surface expression in Lunet N#3 cells. (A) Linear regression of HCVcc (Jc-R2A) infectivity in CD81 chimera expressing cells and CD81-LEL surface expression levels measured as MFI after antibody staining. Regression coefficient and data points for each chimera cell line indicated. Mean±SD of three independent biological replicates used for analysis and shown in the graph.

S4

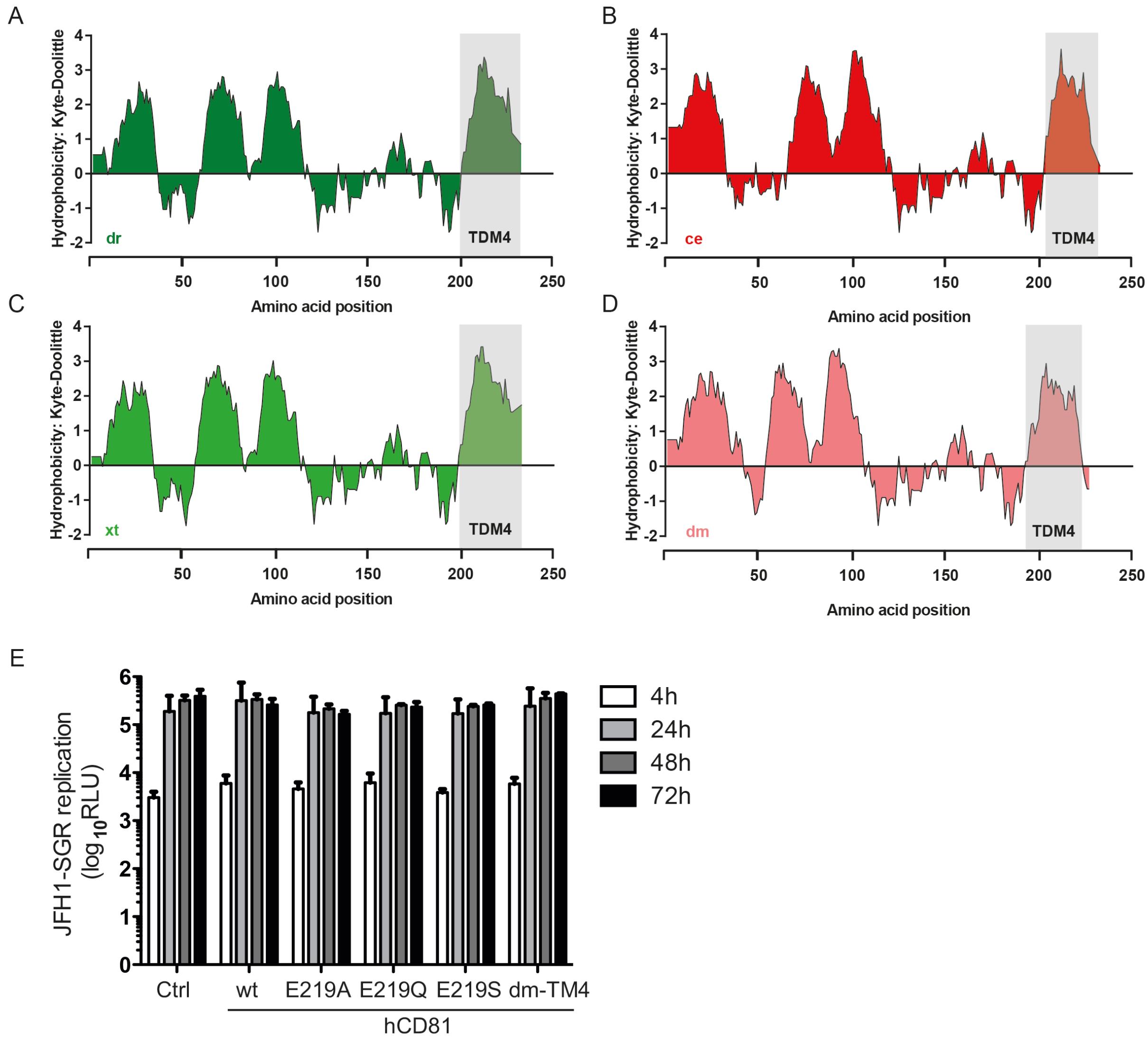

Fig. S4. – Transmembrane domain four residues in dmTSP90F and cdTSP9 critically differ from hCD81. (A-D) Hydrophobicity plots of the hCD81 chimeras with functional backbones (A, C) or non-functional backbones (B, D). (E) Replication assay for TM4 chimeras using an HCV subgenome (JFH-1). Cell lines expressing hCD81 E219 mutants or the hCD81 fly-TM4 chimera were electroporated with JFH-1 subgenomic RNA carrying a Firefly luciferase reporter gene and intracellular viral RNA was quantified as luciferase activity at the indicated timepoint. Mean+SEM of two independent experiments performed in technical triplicates shown.
