# Supplementary material for: CD81 Receptor Regions outside the Large Extracellular Loop Determine Hepatitis C Virus Entry into Hepatoma Cells
Source: Viruses. 2018 Apr 20;10(4):207. doi: 10.3390/v10040207 (PMC5923501; doi:10.3390/v10040207)
Supplement: Supplementary file 1 [file viruses-10-00207-s001.zip › CD81 backbone Supplement/CD81 backbone Supplemental Tables.pdf]

SUPPLEMENTAL TABLES AND REFERENCES

Supplemental Table 1. Residues in hCD81 and dmTSP96F backbones with critical functions.

CD81 residue	CD81 domain	Corresponding residue in dmTSP96F	Function	Reference	conserved in dmTSP96F backbone
C6	cytoplasmic N-terminus	C6	palmitoylation	[1,2]	yes
C9	cytoplasmic N-terminus	C9	palmitoylation	[1,2]	yes
N18	TM1	N18	cholesterol contact, hydrogen bonds	[3]	yes
F21	TM1	F21	cholesterol contact	[3]	yes
N43	small extracellular loop	L43	predicted beta sheet, interaction with LEL	[4]	no
L44	small extracellular loop	n.a.	predicted beta sheet, interaction with LEL	[4]	no
L45	small extracellular loop	n.a.	predicted beta sheet, interaction with LEL	[4]	no
Y46	small extracellular loop	n.a.	predicted beta sheet, interaction with LEL	[4]	no
L47	small extracellular loop	n.a.	predicted beta sheet, interaction with LEL	[4]	no
E48	small extracellular loop	n.a.	predicted beta sheet, interaction with LEL	[4]	no
I64	TM2	V58	cholesterol contact	[3]	hydrophobic
V68	TM2	I62	cholesterol contact	[3]	hydrophobic

V71	TM2	L65	cholesterol contact	[3]	hydrophobic
M72	TM2	I66	cholesterol contact	[3]	hydrophobic
V75	TM2	G69	cholesterol contact	[3]	hydrophobic
F94	TM3	F88	cholesterol contact	[3]	yes
L98	TM3	I92	cholesterol contact	[3]	hydrophobic
L101	TM3	V95	cholesterol contact	[3]	hydrophobic
K116	TM3	K110	salt bridge open conformation	[3]	yes
D117	TM3	D111	salt bridge open conformation	[3]	yes
E219	TM4	S250 (E247)	cholesterol contact, hydrogen bonds	[3]	no
C228	cytoplasmic C-terminus	C259	palmitoylation	[1,2]	yes

Supplemental Table 2. Critical residues in the hCD81 LEL and their function.

**CD81 residue**	**CD81 domain**	**Function**	**Reference**
Q125	LEL	predicted SEL contact	[4]
T149	LEL	predicted CDLN1 contact	[5]
E152	LEL	predicted CDLN1 contact	[5]
T153	LEL	predicted CDLN1 contact	[5]
T153	LEL	predicted SEL contact	[4]
C156	LEL	disulfite bond with C190	[3]

C157	LEL	disulfite bond with C175	[3]
L162	LEL	HCV E2 binding	[6]
T163	LEL	HCV E2 binding	[7]
L171	LEL	HCV E2 binding	[8]
L174	LEL	HCV E2 binding	[8]
C175	LEL	disulfite bond with C157	[3]
I181	LEL	HCV E2 binding	[8]
I182	LEL	HCV E2 binding	[6,8]
N184	LEL	HCV E2 binding	[6]
L185	LEL	HCV E2 binding	[8]
F186	LEL	HCV E2 binding	[7,8]
K187	LEL	HCV E2 binding	[8]
E188	LEL	HCV E2 binding	[8]
D189	LEL	HCV E2 binding	[8]
C190	LEL	disulfite bond with C156	[3]
D196	LEL	HCV E2 binding	[7]
D196	LEL	salt bridge closed conformation	[3]
K201	TM4	salt bridge closed conformation	[3]

Bibliography

1. Delandre, C.; Penabaz, T. R.; Passarelli, A. L.; Chapes, S. K.; Clem, R. J. Mutation of juxtamembrane cysteines in the tetraspanin CD81 affects palmitoylation and alters interaction with other proteins at the cell surface. *Exp Cell Res* **2009**, *315*, 1953–1963.

2. Clark, K. L.; Oelke, A.; Johnson, M. E.; Eilert, K. D.; Simpson, P. C.; Todd, S. C. CD81 associates with 14-3-3 in a redox-regulated palmitoylation-dependent manner. *J Biol Chem* **2004**, *279*, 19401–19406.

3. Zimmerman, B.; Kelly, B.; McMillan, B. J.; Seegar, T. C.; Dror, R. O.; Kruse, A. C.; Blacklow, S. C. Crystal Structure of a Full-Length Human Tetraspanin Reveals a Cholesterol-Binding Pocket. *Cell* **2016**, *167*, 1041–1051.e11.

4. Seigneuret, M. Complete predicted three-dimensional structure of the facilitator transmembrane protein and hepatitis C virus receptor CD81: conserved and variable structural domains in the tetraspanin superfamily. *Biophys J* **2006**, *90*, 212–227.

5. Davis, C.; Harris, H. J.; Hu, K.; Drummer, H. E.; McKeating, J. A.; Mullins, J. G.; Balfe, P. In silico directed mutagenesis identifies the CD81/claudin-1 hepatitis C virus receptor interface. *Cell Microbiol* **2012**, *14*, 1892–1903.

6. Drummer, H. E.; Wilson, K. A.; Poumbourios, P. Determinants of CD81 dimerization and interaction with hepatitis C virus glycoprotein E2. *Biochem Biophys Res Commun* **2005**, *328*, 251–257.

7. Kitadokoro, K.; Bordo, D.; Galli, G.; Petracca, R.; Falugi, F.; Abrignani, S.; Grandi, G.; Bolognesi, M. CD81 extracellular domain 3D structure: insight into the tetraspanin superfamily structural motifs. *EMBO J* **2001**, *20*, 12–18.

8. Chang, C. C.; Hsu, H. J.; Yen, J. H.; Lo, S. Y.; Liou, J. W. A Sequence in the loop domain of hepatitis C virus E2 protein identified in silico as crucial for the selective binding to human CD81. *PLoS ONE* **2017**, *12*, e0177383.
