# Supplementary figures and images for: CD81 Receptor Regions outside the Large Extracellular Loop Determine Hepatitis C Virus Entry into Hepatoma Cells
Source: Viruses. 2018 Apr 20;10(4):207. doi: 10.3390/v10040207 (PMC5923501; doi:10.3390/v10040207)

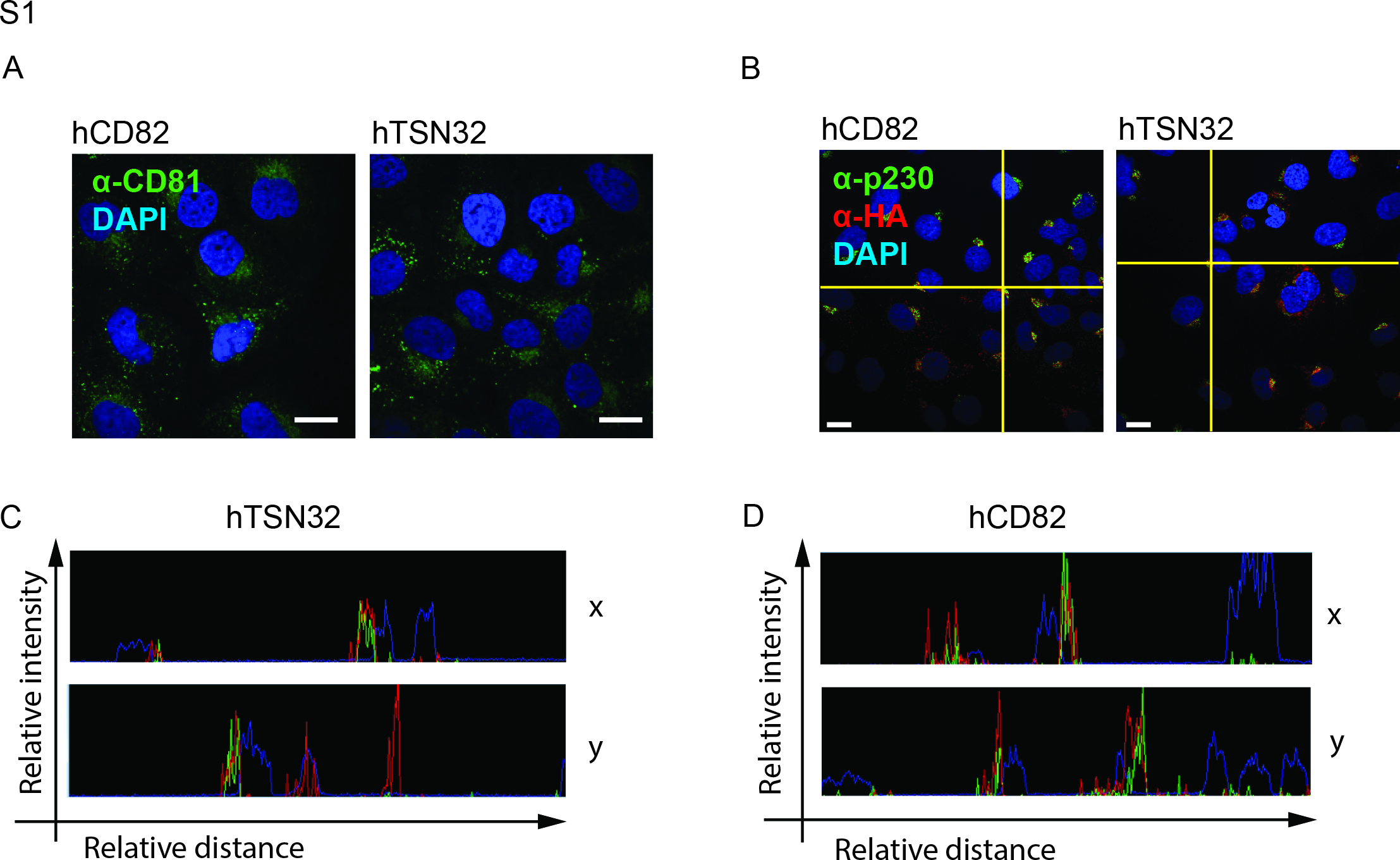

Supplement: Supplementary file 1 [file viruses-10-00207-s001.zip › CD81 backbone Supplement/Figure S1 CD81 backbone.tif]

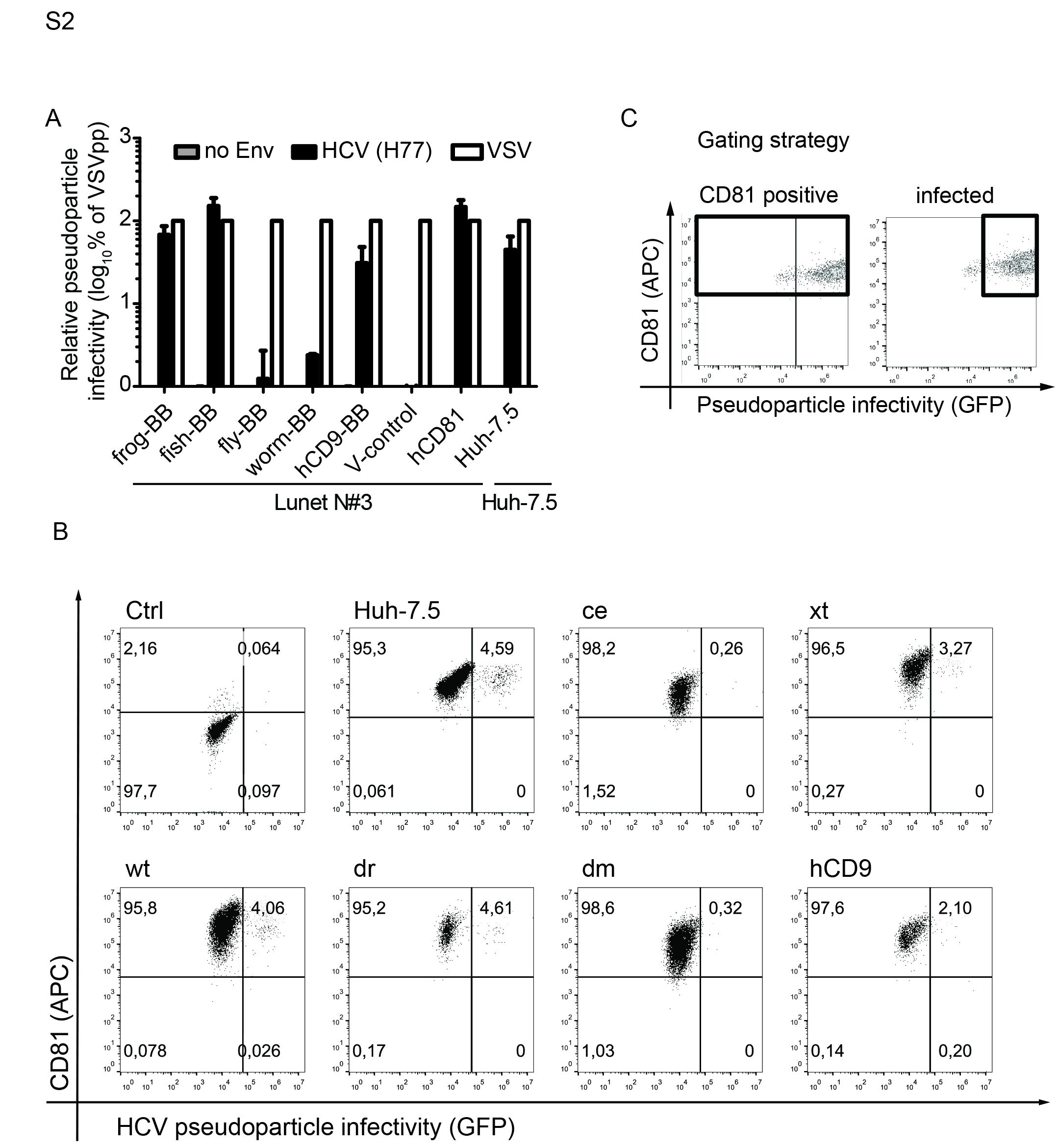

Supplement: Supplementary file 1 [file viruses-10-00207-s001.zip › CD81 backbone Supplement/Figure S2 CD81 backbone.tif]

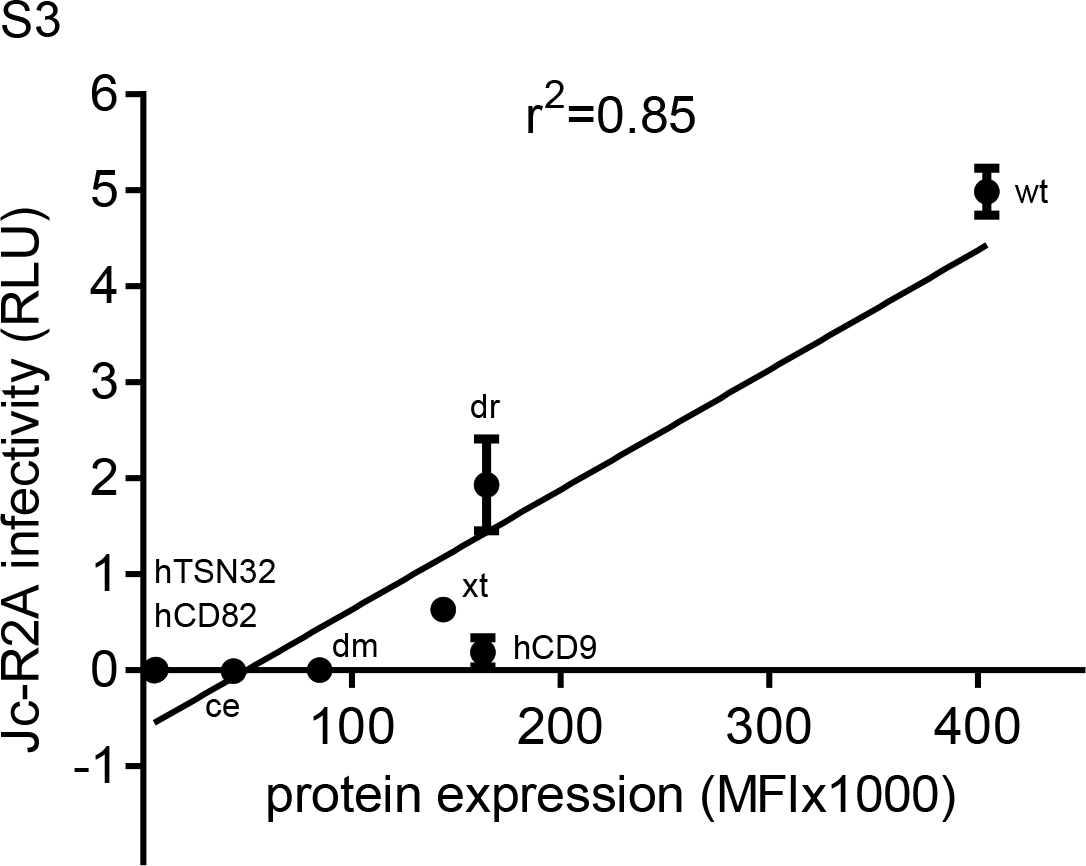

Supplement: Supplementary file 1 [file viruses-10-00207-s001.zip › CD81 backbone Supplement/Figure S3 CD81 backbone.tif]

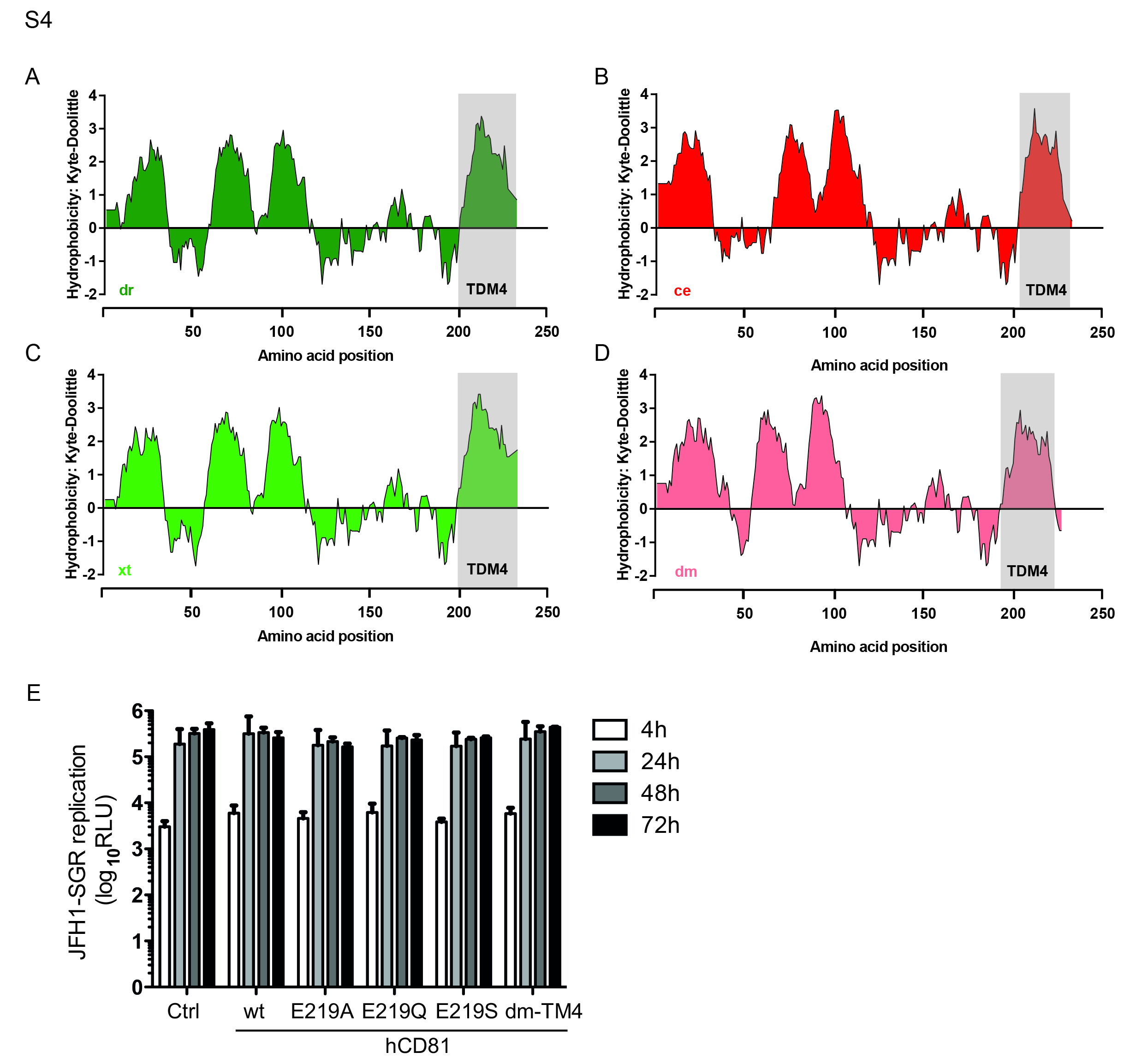

Supplement: Supplementary file 1 [file viruses-10-00207-s001.zip › CD81 backbone Supplement/Figure S4 CD81 backbone.tif]
